# Interface Thermal Resistance in Heterostructures of Micro–Nano Power Devices: Current Status and Future Challenges

**DOI:** 10.3390/nano15161236

**Published:** 2025-08-13

**Authors:** Yinjie Shen, Jia Fu, Fengguo Han, Dongbo Li, Bing Yang, Yunqing Tang

**Affiliations:** 1 School of Mechanical Engineering, Shandong University, Jinan 250061, China; 2112203014@stmail.ujs.edu.cn (Y.S.); fujia@mail.sdu.edu.cn (J.F.); 2 Laboratory of Advanced Design, Manufacturing & Reliability for MEMS/NEMS/OEDS, Jiangsu University, Zhenjiang 212013, China; 3 State Key Laboratory of Advanced Equipment and Technology for Metal Forming, Shandong University, Jinan 250061, China; 4 Key Laboratory of High Efficiency and Clean Mechanical Manufacture of Ministry of Education, Jinan 250061, China; 5 Key National Demonstration Center for Experimental Mechanical Engineering Education, Jinan 250061, China; 6 Shandong Mingde Machinery Co., Ltd., Ji’ning 272071, China; mdkj@mingdejx.com; 7 School of Mechanical and Electrical Engineering, Huainan Normal University, Huainan 232038, China; lidongbo1994@hnnu.edu.cn; 8 Centre for Advanced Laser Manufacturing (CALM), School of Mechanical Engineering, Shandong University of Technology, Zibo 255000, China; yangbingem@sdut.edu.cn; 9 Guangxi Key Laboratory of Manufacturing System & Advanced Manufacturing Technology, Guilin University of Electronic Technology, Guilin 541004, China

**Keywords:** micro-nano power devices, interface thermal resistance, phonon transport, thermal characterization, interface engineering

## Abstract

As micro–nano power devices have evolved towards high frequency, high voltage, and a high level of integration, the issue of thermal resistance at heterointerfaces has become increasingly prominent, posing a key bottleneck that limits device performance and reliability. This paper presents a systematic review of the current state of research and future challenges related to interface thermal resistance in heterostructures within micro and nano power devices. First, based on phonon transport theory, we conducted an in-depth analysis of the heat transfer mechanisms at typical heterointerfaces, such as metal–semiconductor and semiconductor–semiconductor, and novel low-dimensional materials. Secondly, a comprehensive review of current interface thermal resistance characterization techniques is provided, including the application and limitations of advanced methods such as time domain thermal reflection and Raman thermal measurement in micro- and nano-scale thermal characterization. Finally, in response to the application requirements of semiconductor power devices, future research directions such as atomic-level interface engineering, machine learning-assisted material design, and multi-physics field collaborative optimization are proposed to provide new insights for overcoming the thermal management bottlenecks of micro–nano power devices.

## 1. Introduction

**Figure 1:**
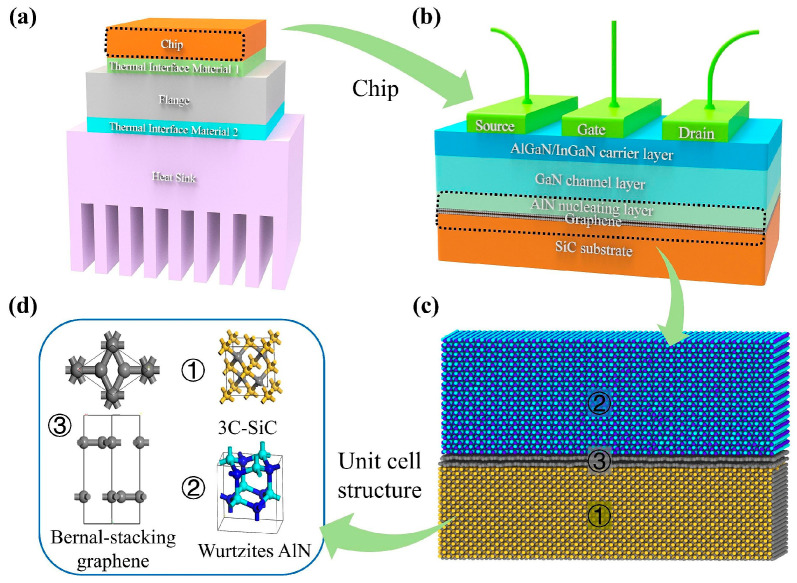
The structures of (**a**) GaN power devices, (**b**) a GaN power chip, (**c**) w-AlN/graphene/3C-SiC, (**d**) 3C-SiC, wurtzite AlN, and AB-stacking graphene [41].

With breakthroughs in semiconductor technology, power devices are moving towards integration at the micro- and nanoscales, resulting in exponential growth in power density [1,2,3,4,5,6]. This has led to increasingly serious thermal management issues. Research shows that at the micro- and nanoscales, interfacial heat dissipation accounts for more than 60% of the total thermal resistance compared to bulk materials [7]. In 1941, Kapitza [8] reported a temperature jump measured near the solid–liquid interface, thereby discovering the interface thermal resistance (ITR), also known as the Kapitza thermal resistance. The interface thermal resistance generated by the large number of heterointerfaces in nanoscale power devices induces severe interface heat dissipation, which significantly impedes heat dissipation and has a major impact on the stability and functionality of the devices [9,10,11,12,13,14,15,16]. Figure 1 shows a schematic diagram of interfacial heat transfer in a typical GaN micro–nano power device. Previous reports have indicated that interfacial thermal resistance dominates the overall thermal resistance at the micro–nano scale [17,18]. Therefore, a comprehensive understanding of interface heat transfer behavior and a systematic revelation of the intrinsic mechanisms of heat carrier interface transfer can guide the development of thermal management in micro–nano power devices. In addition, the widespread application of three-dimensional heterostructure integration technology has further exacerbated the thermal mismatch problem between heterogeneous materials, making interface thermal management a key bottleneck limiting device performance improvement [19,20,21,22]. Furthermore, at the nanoscale, heat transport behavior deviates significantly from classical Fourier’s law, a phenomenon that has been thoroughly confirmed by both experimental and theoretical studies [23,24,25]. The physical origins of this phenomenon include competition between the characteristic length scale and the phonon mean free path, non-equilibrium phonon transport dominance, and temperature discontinuities at heterogeneous interfaces.

At heterogeneous interfaces, the physical essence of ITR originates from the non-equilibrium transport process caused by the combined effects of interface phonon density of states (PDOS) mismatch and interface defect scattering [26,27]. Research on interface heat transfer involves fundamental scientific issues such as multi-physics coupling (electron–phonon–photon interactions) and cross-scale heat transport (from atomic-level phonon states to macroscopic thermal conductivity) [28,29,30]. This is of great significance for improving the theory of heat conduction at the micro- and nanoscales. In addition, precise control of ITR can significantly improve the thermal management capabilities of devices. For example, the interfacial thermal resistance of silicon/graphene/high-entropy alloy heterojunctions can be reduced by 41.47% through interface modification [31]. In recent years, with the development of advanced characterization techniques (such as time domain thermal reflection spectroscopy, Raman thermal measurement, and in situ thermal characterization techniques) and computational simulation methods (molecular dynamics and first-principles calculations) [32,33,34,35,36,37], researchers can measure and predict ITR more accurately and explore methods to regulate it. These advances have not only promoted in-depth basic research, but also provided practical technical approaches for optimizing thermal management in micro–nano power electronic devices. It is worth noting that many reviews on the thermal management of micro–nano devices have been reported previously [2,38,39,40]. However, a comprehensive review of thermal resistance at heterojunction interfaces in micro- and nanoscale power devices is currently lacking, especially at the atomic scale for metal–semiconductor and semiconductor–semiconductor interfaces, as well as novel low-dimensional materials. In addition, existing reviews have mostly focused on surface or simple heterojunction interface heat transfer phenomena, with a lack of systematic summaries of the heat resistance transmission mechanisms at interfaces. Therefore, this review focuses on heat transfer at different types of heterointerfaces, systematically summarizing the intrinsic mechanisms of heat resistance at typical heterostructure interfaces from a phononic perspective.

This paper provides a systematic review of the research progress in interfacial heat transfer in micro–nano power devices, with a focus on three typical heterojunction systems (metal–semiconductor, semiconductor–semiconductor, and novel low-dimensional material heterostructures). The discussion is conducted using a logical framework of ‘mechanism–characterization–regulation’. The specific structure is arranged as follows: Section 2 and Section 3 introduce the physical mechanisms of interface heat transfer modes and experimental measurement progress, respectively. Furthermore, the discussion covers the impact of interface control strategies on interface heat transfer, as well as important factors affecting interface heat transport. Finally, the shortcomings and future challenges of current research on heat transport in interfaces are discussed, and corresponding prospects are presented in Section 4.

## 2. Physical Mechanisms of Interface Thermal Transfer in Heterostructures

Given that phonons are the primary heat carriers in semiconductor devices, extensive research has been conducted on their thermal transport behavior in various nanoscale heterostructures [37,38,42,43]. The breaking of translational symmetry and lattice mismatch can lead to the emergence of new phonon modes at heterointerfaces, namely interface phonon modes [44,45]. Compared with phonon modes in bulk materials, these interface phonon modes exhibit different transport behaviors [28]. They dominate heat transport at micro- and nanoscale interfaces and significantly influence the thermal characteristics of devices. However, early continuous medium hypotheses [46,47,48,49,50,51,52] did not treat materials as discrete lattices, thereby limiting their accuracy in describing detailed phonon modes, as phonon modes manifest as lattice waves. Fortunately, atomic-scale numerical simulation methods such as molecular dynamics (MD), the Boltzmann transport equation (BTE), lattice dynamics (LD), and atomic Green’s functions (AGFs) have significant advantages when studying interface phonon modes in that they accurately capture lattice vibration waves and atomic arrangements near the interface. MD is particularly useful for describing the transmission or scattering of phonons at interfaces in heterogeneous structures, providing different physical perspectives on interface phonon modes and phonon heat transport. This paper will mainly discuss phonon transport theory and interfacial heat conduction in heterostructures from the MD perspective.

### 2.1. Definition of Interface Thermal Resistance at Micro- and Nanoscales

Initially, Fourier used the term ‘external conductivity’ to describe the heat that passes through a surface per unit time, per unit area, and per unit temperature drop [53]. This definition is identical to the modern term interface thermal conductivity (ITC). Unlike the thermal conductivity coefficient (W/mK), ITC has a different unit of length, which is W/m^2^K. Later, Poisson began to study the continuity of heat flux at interfaces [54]:

(1) $$J=\kappa_{1}{\left|\nabla T\right|}_{1}=\kappa_{2}{\left|\nabla T\right|}_{2}=\overset{\cdot }{Q}/A=h_{I}\Delta T$$

where $\kappa_{1}$ and $\kappa_{2}$ are the thermal conductivity coefficients of the two materials, respectively. ${\left|\nabla T\right|}_{1}$ and ${\left|\nabla T\right|}_{2}$ are the temperature gradient moduli evaluated at the interface between the two materials. $\overset{\cdot }{Q}$ is the heat current; A represents the cross-sectional area of the interface. ΔT represents the temperature jump at the interface. $h_{I}$ is the interface thermal conductivity. Therefore, we can define ITR ($R_{I}$) or ITC ($h_{I}$) as follows:

(2) $$R_{I}=\frac{1}{h_{I}}=\frac{\Delta T}{J}$$

**Figure 2:**
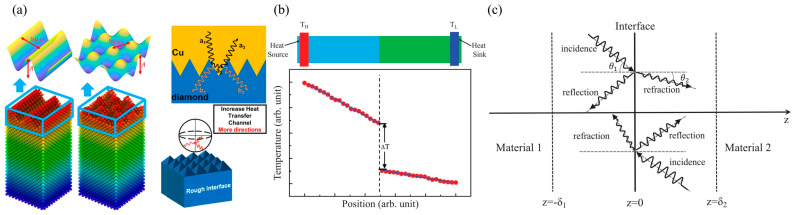
(**a**) Phonon scattering mechanisms at rough interfaces in heterostructures at the nanoscale [55]. (**b**) Schematic diagram of an interface consisting of two different parts and a temperature curve. Here, a sudden temperature discontinuity ΔT can be observed [38]. (**c**) Phonon reflection and refraction on an ideal interface. $\theta_{1}$ and $\theta_{2}$ represent the incident angle and refraction angle on both sides, respectively [38].

Unlike macro interfaces, at the nanoscale, interface morphology or contact area significantly affects ITC or ITR. For example, Wang et al. [55] studied the effect of interface roughness on ITC between copper and diamond through MD simulation and interface thermal measurement experiments. MD simulations found that rough interfaces have higher heat transfer efficiency. Compared with flat interfaces, ITC can be increased by 5.5 times, reaching 133 MW/m^2^K. This is mainly because the roughness of the interface alters the actual contact area between the two materials, thereby affecting the probability of phonon scattering at the interface. The interface scattering mechanism is shown in Figure 2a. Therefore, for heat transfer at the nanoscale interface, ITR is highly dependent on the contact area and geometry near the interface.

In reality, due to lattice mismatch or atomic dislocations, the interface formed by dissimilar materials may not be a sharp plane, as shown in Figure 2b. Therefore, the interface must be extended to a limited area. As shown in Figure 2c, we consider here that the ideal interface plane at z=0 extends to both sides with finite thicknesses $\delta_{1}$ and $\delta_{2}$, respectively. Under the assumption of local thermal equilibrium, the concept of temperature can also be extended to non-equilibrium states. In this case, local temperatures can be defined within a certain area. In atomic-level simulations, the local temperature $T_{l}$ is actually defined by the ensemble-averaged kinetic energy:

(3) $$T_{l}=\frac{\langle \sum_{i=1}^{N}m_{i}v_{i}\cdot v_{i}\rangle }{3Nk_{B}}$$

where N is the number of atoms in the local region, $m_{i}$ and $v_{i}$ are the mass and velocity of the ith atom, respectively, and the brackets indicate the ensemble average. Non-equilibrium molecular dynamics simulations can obtain the non-equilibrium steady-state temperature distribution in the system, as shown in Figure 2b. It can directly capture temperature discontinuities at the interface ΔT. The calculation method for heat flux J is the amount of energy transferred through a unit area per unit time, which can be recorded by the energy injection or extraction rate in the heat source or heat sink. Finally, ITR can be calculated using Equation (2).

### 2.2. Phonon Transport Theory and Interface Heat Conduction Model

MD simulation using classical potential energy is a powerful technique for handling many-body problems at the atomic level. Here, the full-order non-harmonic effect is essentially included in the atomic interaction. Dickey and Paskin [56] used computational simulations to obtain the phonon density of states (PDOS) and gained detailed insights into the phonon properties of solids. PDOS can be obtained by performing a Fourier transform on the velocity autocorrelation function of atoms:

(4) $$\mathrm{PDOS}\left(\omega \right)=\int_{-\infty }^{+\infty }\langle \frac{1}{N}\sum_{i=1}^{N}\vec{v}i\left(t0\right)-\vec{v}i\left(t0+t\right)\rangle e^{-2\pi i\omega t}dt$$

where $v_{i}(t)$ stands for atomic velocity concerning time t; N and ω represent the number of atoms and frequency, respectively. Under thermodynamic equilibrium conditions, the change in atomic velocity $v_{i}(t)$ over simulation time can be obtained from MD simulations.

The vibrational modes of heterogeneous interface phonons are characterized using the phonon coupling coefficient (*S*), which explains the mechanism of interface thermal conductivity changes in heterostructures [57]. The phonon coupling coefficient is calculated from the PDOS. It is calculated from Equation (5).

(5) $$S=\frac{\int_{0}^{\infty }\min\left\{{\mathrm{PDOS}}_{A}\left(\omega \right),{\mathrm{PDOS}}_{B}\left(\omega \right)\right\}d\omega }{\int_{0}^{\infty }\max\left\{{\mathrm{PDOS}}_{A}\left(\omega \right),{\mathrm{PDOS}}_{B}\left(\omega \right)\right\}d\omega }$$

where ${\mathrm{PDOS}}_{A}\left(\omega \right)$ and ${\mathrm{PDOS}}_{B}\left(\omega \right)$ denote the PDOS of material *A* and *B* at ω frequency, respectively, and S represents the phonon coupling coefficient.

The phonon participation rate (PPR) is an effective way to characterize the degree of phonon localization during transmission [58]. The PPR can be further calculated from the PDOS with the expression of Equation (6).

(6) $$\mathrm{PPR}(\omega )=\frac{1}{N}\frac{{\left(\sum_{i}{\mathrm{PDOS}}_{i}{\left(\omega \right)}^{2}\right)}^{2}}{\sum_{i}{\mathrm{PDOS}}_{i}{\left(\omega \right)}^{4}}$$

where *N* indicates the total number of atoms, ω is the frequency, and ${\mathrm{PDOS}}_{i}\left(\omega \right)$ denotes the PDOS of atom *i* at the frequency of interest. The PPR provides detailed information on the localization of each phonon mode.

The degree of localization of a particular phonon localization mode (Λ ∈ PPR < 0.4) is calculated by Equation (7).

(7) $$\phi_{i\alpha ,\Lambda }=\frac{\int_{\Lambda }{\mathrm{PDOS}}_{i\alpha }d\omega }{\frac{1}{N}\sum_{1\leq i\leq N}\int_{\Lambda }{\mathrm{PDOS}}_{i\alpha }d\omega }$$

where a larger value of $\phi_{i\alpha ,\Lambda }$ indicates a stronger localization of mode Λ for i atoms.

To elucidate the contribution of spectral phonons on interfacial thermal conductance, conducted spectral heat current (SHC) analysis utilizing nonequilibrium molecular dynamics simulations. The SHC of frequency-dependent can be expressed as Equation (8). Previous studies have revealed the thermal transport properties of heterostructure interfaces through SHC [59].

(8) J(ω)≈−2tsimuωRe∑i∈A∑j∈B∑α,β∈x,y,zImv^iα(ω)∗Kijαβv^jβ(ω)

where ${\hat{v}}_{i}^{\alpha }(\omega )$ and ${\hat{v}}_{j}^{\beta }(\omega )$ are the Fourier transform atomic velocities of atoms *i* and *j* in region A and region B, respectively. $K_{ij}^{\alpha \beta }$ denotes the matrix of interfacial force constants determined by the finite difference method, and $t_{\mathrm{simu}}$ denotes the force–velocity correlation time. Based on Fourier’s law, the spectral ITC can be expressed as $G\left(\omega \right)$ by

(9) $$G\left(\omega \right)=\frac{\left|J\left(\omega \right)\right|}{A\Delta T}$$

where *A* is the interfacial overlapping area of heterogeneous structures. ΔT is the interface temperature difference.

In MD numerical simulations of heat transfer at heterogeneous interfaces, commonly used methods include non-equilibrium molecular dynamics (NEMD) and transient thermal pulse methods. For example, Wang et al. [60] constructed a model of partially stacked graphene sheets with random vacancies and used the NEMD method to study the effects of vacancy coverage, stacking length, and stacking form on the heat transfer coefficient. The NEMD method involves setting up heat sources and cold sources at both ends of the simulated system. The temperature difference between the hot bath and the cold bath drives heat flow from the hot end to the cold end, forming a steady-state temperature gradient. The linear extrapolated temperature difference values on both sides of the interface are extracted from the temperature profile. The definition of interfacial thermal conductivity calculated using the NEMD method is the ratio of heat flux J to interfacial temperature difference ΔT, expressed by Equations (10) and (11).

(10) G=J/ΔT

(11) $$J=\frac{1}{V}\langle \sum_{i}v_{i}E_{i}+\frac{1}{2}\sum_{i\neq j}r_{ij}\left(F_{ij}\cdot v_{i}\right)\rangle$$

Among them, G represents the interface thermal conductivity, $E_{i}$ represents the atomic energy, and $F_{ij}$ represents the interatomic force.

**Figure 3:**
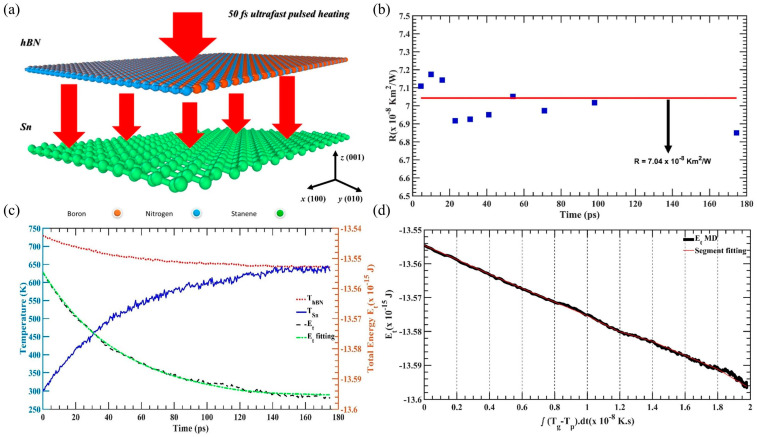
Interface thermal conductivity numerical simulation method [61]. (**a**) Schematic diagram of the transient thermal pulse method calculation of Sn/h-BN heterostructure ITR. (**b**) Fluctuations in ITR under different relaxation times. (**c**) Changes in temperature and energy during transient heating. (**d**) Total energy of h-BN layers as a function of ∫ΔTdt.

Compared with the NEMD method, the transient thermal pulse method is more suitable for calculating the interface thermal properties of two-dimensional heterostructures. For example, Das et al. [61] used the transient thermal pulse method to analyze the interface thermal resistance of Sn/h-BN heterostructures, as shown in Figure 3a–d. The results show that at room temperature, the ITR value of 30 × 10 nm^2^ Sn/h-BN is approximately 7 × 10^−8^ Km^2^/W. It is worth noting that the transient thermal pulse is achieved by applying a localized and short-duration non-equilibrium energy perturbation to simulate the thermal pulse excitation process in the experiment, thereby studying the thermal transport response of the system (especially micro–nano scale materials or interfaces). The ITC is obtained using the temperature and energy data according to Equations (12) and (13), utilizing the transient thermal pulse method.

(12) $$E_{A}=-S_{area}\cdot K\int \left(T_{A}-T_{B}\right)dt+E_{0}$$

(13) $$\frac{\partial E_{A}}{\partial t}=-S_{area}\cdot \left(T_{A}-T_{B}\right)\cdot K$$

where $S_{area}$ denotes the interfacial overlap area, K represents the ITC, and $E_{A}$ represents the material *A* layer energy. $T_{A}-T_{B}$ denotes the temperature difference between the material *A* and *B* layers.

### 2.3. Thermal Transport Properties of Typical Metal–Semiconductor Interfaces

Metal–semiconductor interfaces are core components of modern electronic devices and are widely found in key devices such as transistors, photodetectors, and power modules [62,63,64,65,66]. Traditional thinking suggests that heat conduction at metal–semiconductor interfaces mainly depends on electron–phonon coupling processes. However, recent studies have pointed out that phonon inelastic scattering is the main carrier of heat transport at the metal–semiconductor interface [9,28,44,67,68,69]. For example, Xu et al. [67] demonstrated that the thermal conductivity of a metal–semiconductor interface (i.e., Al/Si interface) at room temperature is approximately 350 MW/m^2^K, which is 1.43 times the harmonic limit. The phonon–phonon coupling provides the main channel for heat transfer at the Al/Si heterointerface. This indicates that fully exploring the phonon transport behavior at the interface and revealing the underlying mechanisms are crucial for the thermal management of metal–semiconductor-based micro–nano devices.

**Figure 4:**
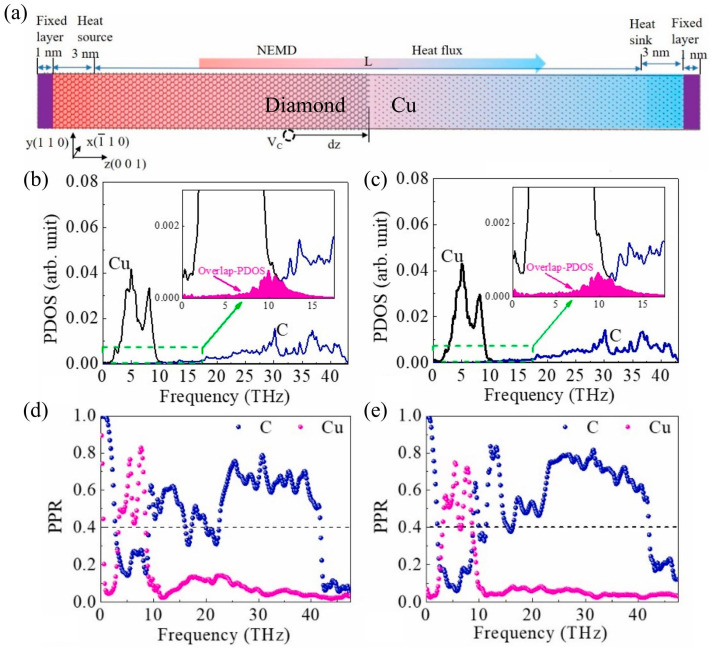
NEMD numerical simulation of thermal conductivity and interface phonon mode analysis at the Cu (111)/diamond heterojunction interface [73]. (**a**) Schematic diagram of NEMD calculation of ITC. (**b**,**c**) are PDOS distribution diagrams for C vacancy concentrations of 0.75% and 1.5%, respectively. (**d**,**e**) are distribution diagrams for PPR with C vacancy concentrations of 0.75% and 1.5%, respectively.

Phonon heat transfer at metal–semiconductor heterointerfaces has been extensively studied [70,71]. For example, Adnan et al. [70] used machine learning molecular dynamics (MLMD) to predict the ITC of some metal–diamond heterointerfaces with promising applications. The predicted TBCs of relaxed Al, Mo, Zr, and Au–diamond interfaces are approximately 284, 93, 30, and 40 MW/m^2^K, respectively, after quantum corrections. Table 1 summarizes the numerical simulation values of the interfacial thermal conductivity of typical metal–semiconductor heterostructures. Among them, research on interface heat transport in copper-based heterostructures widely used in power devices has been reported in a series of studies [34,65,72]. For example, Liao et al. [62] used non-equilibrium molecular dynamics to elucidate the mechanism by which the two-dimensional sinusoidal surface roughness structure affects the thermal conductivity of the copper/diamond interface. PDOS analysis shows that the rough interface significantly improves the phonon–vibration coupling between copper and diamond, further enhancing the thermal transfer capacity of the copper/diamond interface. Cai et al. [72] used MD simulations to study changes in thermal resistance at the interface of a three-layer heterostructure consisting of diamond/copper/carbon nanotubes after thermal cycling. PDOS results indicate that the increase in load temperature intensifies the low-frequency phonon density distribution. Similarly, Wu et al. [73] reported the effect of carbon vacancies on the ITC of copper/diamond heterostructures using the same simulation method. Research shows that carbon vacancies significantly enhance interfacial heat transport. For the copper (111) surface, the ITC increases from 37.98 MWm^−2^ K^−1^ to approximately 177 MWm^−2^ K^−1^. Furthermore, as can be seen from the analysis of PDOS and PPR in Figure 4, the increase in ITC induced by carbon vacancies is mainly due to the enhancement of interface phonon modes. It is worth noting that their research reveals the evolutionary behavior of interfacial heat transport from the perspective of changes in the phonon mode density distribution or transmission caused by interfacial lattice vibrations. This approach of exploring the origin of interface phonon vibration modes to explain the intrinsic mechanism of ITC is equally applicable to other metal-based heterointerfaces. For example, Feng et al. [44] studied heat transfer at the silicon/germanium interface through MD modal analysis and clarified that the interface phonon modes act as an interface bridge to produce a bridging effect, enhancing inelastic phonon transport. Surprisingly, due to the bridging effect, the contribution of optical phonon modes to interfacial heat conduction is equal to or greater than that of acoustic modes. Shen et al. [31] used the transient thermal pulse method to numerically calculate the interfacial thermal conductivity of HEA/Gr/Si sandwich heterostructures. Through a comprehensive analysis of phonon density of states, phonon coupling coefficients, phonon participation rates, and phonon coupling spectrum decomposition, the role of substrate morphology in interfacial heat transport was elucidated. It is worth noting that research on the interfacial heat transfer behavior of heterostructures in novel high-entropy alloy systems remains limited, particularly from the perspective of exploring the intrinsic mechanisms of heat transfer at high-entropy alloy-based heterointerfaces through the transmission of phonon heat carriers.

Currently, numerical simulation studies of typical metal–semiconductor interface thermal resistance at the micro- and nanoscales still face several key challenges and limitations. Molecular dynamics simulations rely on empirical potential functions (such as Lennard-Jones, MEAM, EAM, etc.), but existing potential functions do not adequately describe the non-harmonic interactions at metal–semiconductor interfaces (such as Au/Si and Al/GaN), resulting in significant deviations in the prediction of interface thermal conductivity. In addition, the results obtained using different numerical simulation methods for the same heterogeneous structure vary greatly, making it difficult to form a systematic quantitative analysis.

### 2.4. Thermal Transport Properties of Typical Semiconductor–Semiconductor Interfaces

Another common heterostructure in micro–nano power devices is the semiconductor–semiconductor form [77,78,79,80,81,82]. The thermal transport characteristics of semiconductor–semiconductor interfaces directly affect device performance, reliability, and service life, especially in high power density applications. From a physical mechanism perspective, heat transport at semiconductor–semiconductor interfaces is primarily dominated by phonon transport processes, which involve complex multiscale physical phenomena. At the atomic scale, phonon density mismatch at the interface is the main factor causing thermal resistance. Taking the GaN/AlN interface as an example [83], the phonon density of GaN is mainly distributed in the 20–25 THz range, which differs significantly from that of AlN (mainly distributed in the 0–12 THz range). This mismatch causes a significant decrease in the phonon transmission rate at the interface. At the nanoscale, interface defects (such as dislocations, doping, etc.) further enhance phonon scattering [84,85,86]. These complex physical mechanisms make the study of heat transport at semiconductor–semiconductor interfaces a cutting-edge topic at the intersection of condensed matter physics and thermal science. Table 2 summarizes the numerical simulation values of the interfacial thermal conductivity of typical semiconductor–semiconductor heterostructures. Compared with metal–semiconductor structures, the majority of carriers in interface heat transport in semiconductor–semiconductor heterostructures are phonon modes. As previously reported, the interface thermal conductivity of the Ga_2_O_3_/diamond heterostructure improved from 46.1 ± 2.3 to 60.9 ± 3.0 MW/m^2^K, primarily due to enhanced phonon transport at the interface, resulting from increased participation of low-frequency phonons [87].

**Figure 5:**
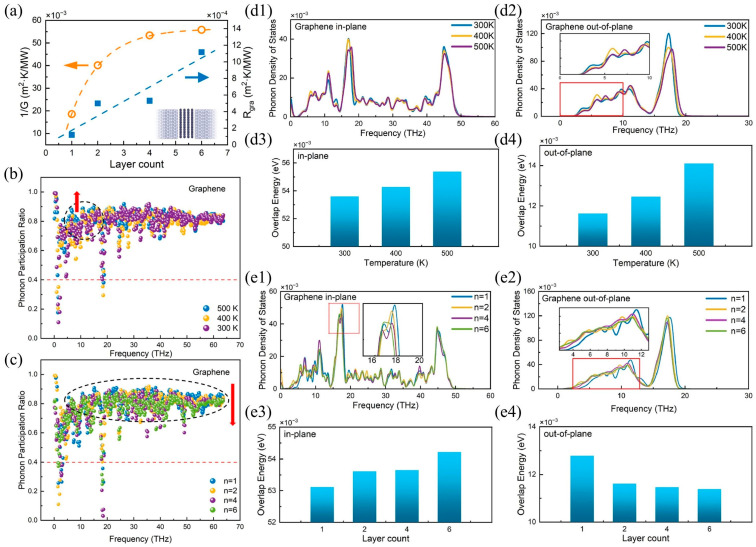
Phononic mechanism analysis of thermal conductivity at the interface of diamond/multi-layer graphene heterojunctions [89]. (**a**) Trends in interface thermal resistance and their fitting curves. (**b**) PPR of graphene layers at different load temperatures. (**c**) PPR of graphene layers at different layer numbers. The comparison of PDOS for graphene: (**d1**) in-plane direction and (**d2**) out-of-plane direction. The interfacial overlap energy: (**d3**) in-plane direction and (**d4**) out-of-plane direction. The PDOS of graphene as a function of graphene layer count: (**e1**) in-plane modes and (**e2**) out-of-plane modes. The interfacial overlap energy: (**e3**) in-plane direction and (**e4**) out-of-plane direction.

In addition, the coupling degree of low-frequency phonons at the interface dominates heat transfer at the interface, especially for semiconductor–semiconductor heterostructures [88]. For example, Liu et al. [89] studied the factors affecting heat transfer at the interface of diamond/multi-layer graphene heterostructures based on the NEMD method. PDOS, interface overlap energy, and PPR reveal the driving effect of low-frequency phonon coupling at 2–20 THz on interface heat conduction, as shown in Figure 5. Yu et al. [90] used full-scale molecular dynamics to significantly alter the ITC of GaN/Diamond from 200 MW/m^2^K to 230 MW/m^2^K through external mechanical loading. This was mainly due to the mechanically adjustable interface morphology and the influence of low-frequency phonon resonance at 0–20 THz. Rajabpour et al. [91] calculated the ITC between silicon and diamond using an efficient machine learning (ML) interatomic potential trained on density functional theory (DFT) data. New frequencies in the low-frequency range of 8–12 THz appeared on the interface, corresponding to local phonon modes, which were not present in the bulk region. Based on the cumulative ITC, these phonon modes make a significant contribution to the ITC of the silicon/diamond heterostructure, with approximately 40% of the total ITC belonging to this frequency range. Furthermore, Liu et al. [92] confirmed that out-of-plane phonons dominate heat transfer at the interface of semiconductor–semiconductor heterostructures. In diamond/graphene heterostructures, graphene defects cause a shift in the PDOS of graphene, resulting in changes in the overlap energy. The overlap energy of face-external phonons is consistent with the ITC change, confirming its dominant role. Despite these advances, research into heat transport at the semiconductor–semiconductor interface still faces many challenges. In terms of fundamental theory, how to establish a theoretical model that can uniformly describe heat transport behavior from the quantum scale to the macroscopic scale remains an open question. Existing phonon transport models still have limitations in explaining certain experimental phenomena, especially for strongly anisotropic heterostructures.

The numerical simulation studies of thermal resistance at semiconductor–semiconductor interfaces (such as Si/Ge, GaN/AlN, SiC/SiO_2_, etc.) still face the following key limitations, which constrain their predictive accuracy and practical application value. Common potential functions (such as Tersoff, S-W, AIREBO, etc.) do not adequately describe the anharmonic interactions at hetero-semiconductor interfaces (such as SiC/GaN), leading to errors in the calculation of phonon scattering rates. Long-range Coulomb interactions at polar semiconductor interfaces (such as GaN/AlN) are difficult to accurately simulate in classical molecular dynamics. Furthermore, actual interfaces contain dislocations, oxide layers, or roughness, but simulations often assume perfect lattice matching, underestimating scattering effects.

**Table 2:** The calculation results of ITC for typical semiconductor–semiconductor heterostructures at room temperature.

Typical Heterostructures	Computational Method	Simulated Values of ITC
Si/diamond	MLMD	110–140 MW/m^2^K [91]
Si/diamond	NEMD	260 MW/m^2^K [93]
crystal-SiC/amorphous-SiC	NEMD	1820 MW/m^2^K [1]
Ga_2_O_3_/diamond	NEMD	46.1 ± 2.3 MW/m^2^K [87]
*w*-AlN/3C-SiC	TTP	43.36 MW/m^2^K [94]
GaN/multilayer graphene	NEMD	88.1 ± 5.9 MW/m^2^K [85]
GaN/AlN	NEMD	625 MW/m^2^K [83]

Notice: MLMD represents the molecular dynamics of machine learning potential. TTP represents the transient thermal pulse method.

### 2.5. Thermal Transport Properties of Novel Low-Dimensional Heterointerfaces

**Figure 6:**
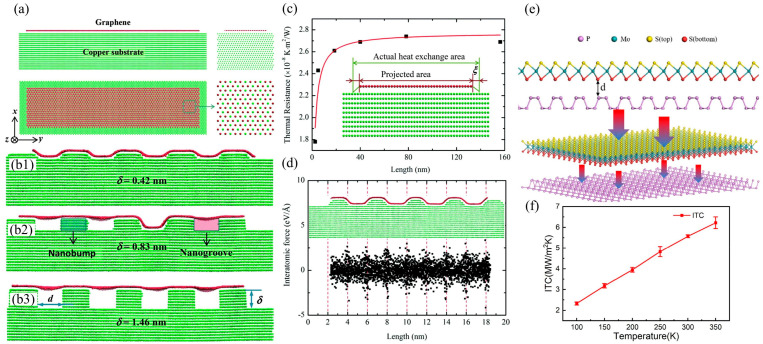
Heat transfer at the interface of a van der Waals heterostructure. (**a**–**d**) The effect of nano-engineered modifications on the Cu substrate on interfacial heat transfer in Cu/graphene van der Waals heterostructures [102]. (**e**,**f**) show the numerical simulation of the ITC at the BP/MoS_2_ van der Waals heterostructure interface using the thermal pulse method [103].

With the rapid development of nanotechnology and two-dimensional materials, low-dimensional heterostructures have become a hot topic of research in the interdisciplinary field of condensed matter physics, materials science, and thermal science due to their unique physical properties and broad application prospects [95,96,97]. Compared with traditional materials, low-dimensional heterointerfaces exhibit many novel thermal transport properties, such as quantum confinement effects, interface phonon coupling, and enhanced near-field thermal radiation [98,99,100], which provide new ideas for the innovation of thermal management technologies. New low-dimensional materials are represented by graphene, hexagonal boron nitride, molybdenum disulfide, phosphorene, and silicene [101]. The heterostructures formed by these materials can be mainly divided into two types: in-plane covalently bonded heterostructures and interlayer van der Waals force heterostructures. Table 3 summarizes the numerical simulation values of the interfacial thermal conductivity of novel low-dimensional material heterostructures. The statistical results in the table show that low-dimensional heterostructures with in-plane covalent bonding have higher ITC values than van der Waals heterostructures. There have been numerous reports on interlayer van der Waals force heterostructures, which are relatively common. For example, Hong et al. [102] used the transient thermal pulse method to calculate the interfacial thermal resistance of the Cu/Graphene heterostructure to be approximately 2.7 × 10^−8^ Km^2^/W, and effectively promoted heat transfer at the Cu/Graphene interface by modifying nano-grooves of different depths on the Cu substrate, as depicted in Figure 6a–d. This is mainly because the suspended region of graphene is pulled towards the substrate by attractive interatomic forces, thereby generating high local pressure in the top region of the nanocolumn, which enhances the thermal coupling between graphene and Cu and reduces the interface thermal resistance. The results in Figure 6e,f indicate that Wu et al. [103] also used the thermal pulse method to numerically study the effect of different vacancy defects on the ITC of BP/MoS_2_ van der Waals heterostructures. The results show that its ITC is 5.572 MW/m^2^K at room temperature, and when the load temperature rises from 100 K to 350 K, the ITC increases by 167%. In addition, it was found that ITC is closely related to the concentration of Mo vacancy defects. Liu et al. [104] numerically simulated the ITC of graphene/silene van der Waals heterostructures, and the ITC of the original graphene/silene bilayer at room temperature was 11.74 MW/m^2^K. In addition, the study found that interface coupling strength and hydrogenation of graphene layers can effectively improve ITC. Zhang et al. [105] calculated the interfacial thermal conductivity of the MoS_2_/amorphous silica heterostructure by fitting the transient temperature decay. Under the influence of the interlayer van der Waals binding energy, the interfacial thermal conductivity between the single-layer MoS_2_ and the amorphous silica substrate gradually began to saturate and reached a maximum of 148 MW/m^2^K, which was attributed to substrate-induced local phonons. For low-dimensional heterostructures with in-plane covalent bonding, they generally have higher ITC. For example, Li et al. [106] used reverse non-equilibrium molecular dynamics (RNEMD) to calculate the ITC of graphene/graphane heterostructures with different configurations of in-plane covalent bonds. The results showed that the ITC of zigzag-graphene/graphane reached 2.2 GW/m^2^K at room temperature, which was higher than that of armchair-graphene/graphane (1.7–1.9 GW/m^2^K). Liu et al. [107] utilized the NEMD method to numerically simulate the ITC of in-plane graphene/MoS_2_ heterostructures, which was found to be 0.225–0.25 GW/m^2^K. Each Mo-C bond at the interface acts as an independent thermal channel, and the thermal conductivity at the interface can be regulated by controlling the concentration of Mo vacancies at the interface. In addition, in-plane covalently bonded heterostructures often exhibit thermal rectification effects. For example, Chen et al. [108] discovered a significant thermal rectification phenomenon in graphene/h-BN through NEMD research. The observed phenomenon can be attributed to the resonance effect of out-of-plane phonon modes in graphene and h-BN domains in the low-frequency region. Similarly, Li et al. [106] utilized gradient hydrogenation to impart similar thermoelectric properties to graphene/graphene nanoribbons while eliminating dependence on chirality and length. The proposed gradient hydrogenation technology can be used for length-insensitive thermal diodes and has practical application value.

**Figure 7:**
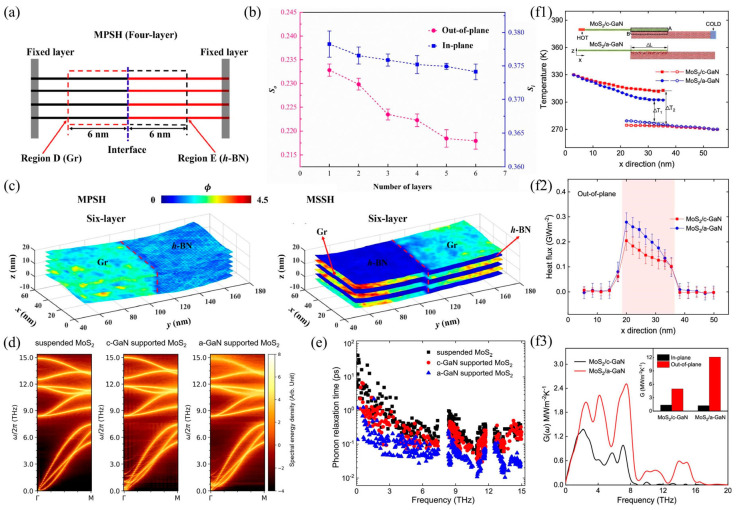
Phononic mechanism analysis of heat transfer at heterointerfaces in low-dimensional materials. (**a**) Schematic diagram of multi-layer Gr/h-BN van der Waals heterostructure [58]. (**b**) The phonon coupling coefficient of in-plane and out-of-plane phonons in Gr/h-BN heterostructures varies with the number of layers [58]. (**c**) Spatial distribution map of phonon localization in multilayer Gr/h-BN van der Waals heterostructures [58]. (**d**) Spectral energy density of suspended MoS_2_ and MoS_2_ supported by c-GaN and a-GaN [59]. (**e**) MoS_2_ phonon relaxation time [59]. (**f1**–**f3**) The temperature curves, out-of-plane heat flux distribution, and atomic vibration direction decomposition spectra ITC of MoS_2_/GaN, respectively [59].

For low-dimensional heterostructures, the intrinsic physical mechanism of interfacial heat transfer behavior can also be analyzed using phonon transport theory. For example, Liang et al. [58] constructed graphene/hexagonal boron nitride (Gr/h-BN) heterostructures with various configurations through van der Waals interactions and used phonon vibration spectra, phonon participation rates, and phonon localization spatial distributions to explore the physical mechanisms behind the phenomenon of interface heat transfer changes. The results shown in Figure 7a–c indicate that the interlayer phonon coupling strength of Gr/h-BN is strongly correlated with ITC. Furthermore, the spatial distribution of phonon localization modes in different heterogeneous structures shows that Gr domains are the main bottleneck in interfacial phonon heat transfer channels. Wang et al. [59] demonstrated through MD simulations that an atomic-scale amorphous thin layer on the substrate surface can significantly improve the ITC of the 2D-MoS_2_/3D-GaN van der Waals interface, increasing it from a maximum of 7 MW/m^2^K to 28 MW/m^2^K. Analysis of the phonon physics mechanism indicates that this enormous ITC enhancement is attributed to the increased low-frequency phonon density and channel at the interface and the enhanced interface phonon coupling, as shown in Figure 7f. Analysis of Figure 7d,e indicates that the slight surface fluctuations and increased diffuse interface scattering of MoS_2_ promote the transfer of energy from in-plane phonons to out-of-plane phonons in MoS_2_, which then transfer to the substrate, thereby promoting interfacial phonon transport. In addition, Ni et al. [109] demonstrated through MD simulations that in graphene/h-BN heterostructures with in-plane covalent bonding, the composition gradient interface can be regulated by phonon localization to modulate heat transfer in the heterostructure.

Despite significant research progress, there are still many key scientific issues regarding low-dimensional heterointerface heat transport that need to be addressed. At the fundamental theoretical level, how to establish a universal multi-body interaction model that uniformly describes electron–phonon–photon coupling effects remains a major challenge facing theoretical physics. In addition, the large-scale controllable integration of low-dimensional materials and precise control of interface quality are also bottleneck issues that constrain practical applications. Addressing these challenges requires deep interdisciplinary integration across physics, materials science, engineering, and other fields.

Numerical simulations of interfacial thermal resistance in low-dimensional heterostructures (such as graphene/h-BN, MoS_2_/WS_2_, carbon nanotubes/polymers, etc.) have made significant progress in recent years, but several fundamental challenges remain. Most studies directly apply the phonon transport model of bulk materials, ignoring the boundary scattering enhancement effects specific to low-dimensional systems (such as the edge phonon localization effect in graphene). The interlayer coupling potential function at the van der Waals interface still relies on simple models such as Lennard-Jones, which cannot describe the phonon mode reconstruction caused by the twist angle. In addition, phonon quantization in the thickness direction of two-dimensional materials significantly alters the phonon density of states, but existing simulations still use the continuous medium approximation, which results in numerical deviations from actual heat transport behavior.

## 3. Characterization Techniques of Interface Thermal Resistance

In recent years, breakthrough progress has been made in micro–nano interface thermal conductivity characterization technology, mainly in three dimensions: spatial resolution, temporal resolution, and measurement accuracy. In terms of spatial resolution, FET-Raman technology has achieved nanometer-level spatial resolution. For example, frequency-energy transfer state-resolved Raman spectroscopy (FET-Raman) can be used to extract the true interfacial thermal resistance (determined by phonon transport) [110,111]. In the field of time resolution, femtosecond laser pump-probe technology has improved time resolution to the 100 fs level, enabling the capture of transient heat transport processes at interfaces [112]. In terms of measurement accuracy, the time domain thermal reflectometry (TDTR) method controls the uncertainty of interface thermal conductivity measurements to within ±10% through phase-sensitive detection [113]. Notably, in 2025, Liu et al. [114] reported on electron thermal microscopy technology, which developed electron microscopy techniques for visualizing phonon transport. By constructing a temperature gradient in situ in a scanning transmission electron microscope and combining it with an electron energy loss spectroscopy temperature measurement method, they achieved the first-ever measurement of temperature fields and interfacial thermal resistance at the nanoscale. Additionally, by simultaneously measuring the phonon density of states and population near the interface at the nanoscale, they elucidated the microscopic mechanism of phonon transport across interfaces involving interfacial modes. These advances in micro–nano interface thermal conductivity characterization techniques provide critical support for the development of new thermal management materials and devices, as fully demonstrated in the measurement of heat transfer at interfaces in heterostructures.

### 3.1. Time Domain Thermal Reflection Method (TDTR)

**Figure 8:**
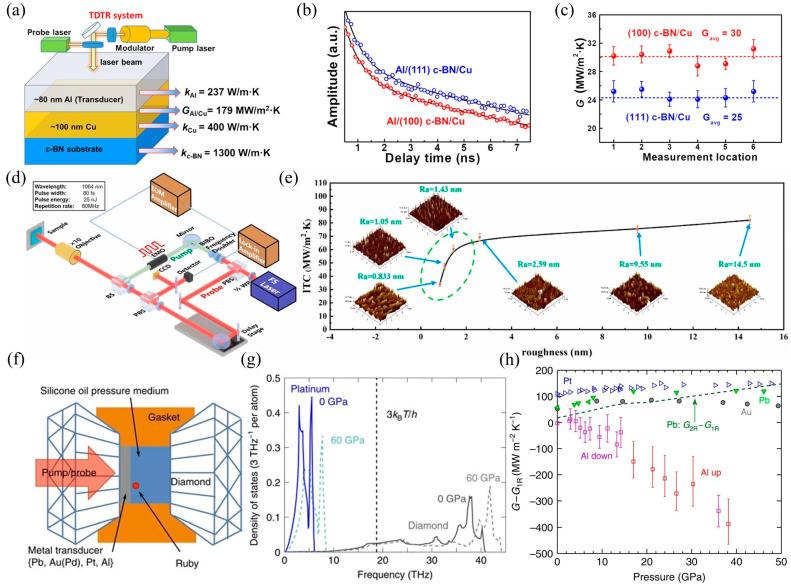
TDTR interface thermal conductivity measurement. (**a**) Schematic diagram of TDTR measurement of c-BN/Cu heterostructure ITC [115]. (**b**) Fitted curve of the TDTR signal during measurement [115]. (**c**) TDTR measurement results of (100) c-BN/Cu and (111) c-BN/Cu heterostructures [115]. (**d**) Schematic diagram of TDTR measurement of Diamond/Cu heterostructure ITC [113]. (**e**) TDTR measurement results of ITC under different interface roughness conditions [113]. (**f**) Schematic diagram of TDTR measurement of ITC in metal/diamond heterostructures under medium and high voltages [68]. (**g**) High pressure increases the maximum phonon frequency of metals and their overlap with the phonon density of states of diamond [68]. (**h**) Excess thermal conductance [68].

TDTR is widely applicable to complex structural systems and can analyze the thermal resistance distribution of each layer and interface in multi-layer heterogeneous structures [68,113,115,116,117,118,119]. Its advantage is that it can measure thermal conductivity and interfacial thermal resistance simultaneously. By fitting the time/frequency response of the heat reflection signal, it is possible to infer the bulk thermal conductivity, interfacial thermal resistance, and even the heat capacity of the material, thereby reducing the cumulative error of multi-step measurements [120,121]. For example, Chen et al. [115] used TDTR technology to study the relationship between interface bonding and interface characteristics, as illustrated in Figure 8a–c. The results indicate that the TDTR-measured ITC value (30 MW/m^2^K) of the (100) c-BN/Cu interface is approximately 20% higher than that of the (111) c-BN/Cu interface (25 MW/m^2^K). This is because the (100) c-BN/Cu interface exhibits higher interfacial bonding strength, thereby demonstrating superior interfacial phonon transport capability. Similarly, Cui et al. [113] measured the ITC of copper/diamond heterostructures using TDTR technology and studied the effect of the roughness of the <010> boron carbide interface on the ITC, as shown in Figure 8d,e. Research shows that when the roughness of the intermediate layer of boron carbide increases from 0.833 nm to 14.5 nm, the ITC increases by approximately 140%, from 33.9 MW/m^2^K to 82.1 MW/m^2^K. Hohensee et al. [68] reported TDTR technology measurements of metal/diamond interface thermal conductance up to 50 GPa in the diamond anvil cell (DAC) for Pb, Au_0.95_Pd_0.05_, Pt, and Al films deposited on type 1A natural [100] and type 2A synthetic [110] diamond anvils. As shown in Figure 8f–h, in all cases, the interface thermal conductivity increases slightly or saturates to similar values under high pressure. Li et al. [122] measured the interface thermal conductivity of GaN/AlN heterostructures using TDTR experiments and achieved 320 MW/m^2^K at room temperature using ultra-fast optical technology and sensitivity checks. The above research shows that TDTR can measure the interfacial thermal conductivity of various materials, including metals, semiconductors, insulators, and polymers, and is particularly effective for materials with high thermal conductivity (such as the c-BN/Cu interface) or low interfacial thermal resistance (such as the metal/semiconductor interface). However, when measuring interface thermal conductivity using TDTR, metal transducers (such as Al films) need to be deposited to enhance the thermal reflection signal, which may introduce external interface effects that affect the actual interface thermal conductivity value of the sample.

### 3.2. Raman Interface Thermal Measurement Technology

**Figure 9:**
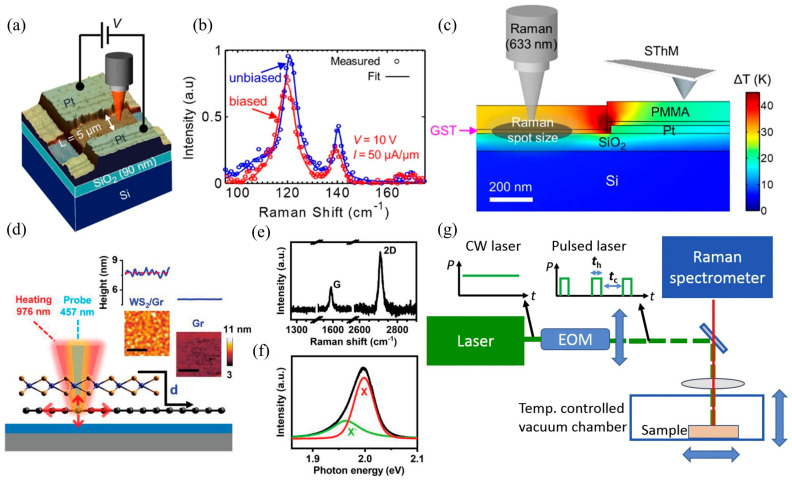
Raman thermal measurement technology applied to interface thermal conductivity measurement. (**a**) Schematic diagram of Raman measurement of ITC in heterostructures [124]. (**b**) Measurement of Raman spectra and fitting of Raman spectrum curves at the channel center GST under conditions of electric bias (red: V = 10 V, I = 0.5 mA) and no bias (blue) [124]. (**c**) Analog cross-sectional temperature distribution near the contact point of the device [124]. (**d**) Experimental setup for Raman measurement of heat transfer characteristics at the interface of WS_2_/Gr heterostructures [125]. (**e**) Raman spectroscopy of graphene in WS_2_/Gr heterostructure measurements [125]. (**f**) Photoluminescence spectrum of WS_2_ [125]. (**g**) Schematic diagram of a completely non-contact laser flash Raman testing method [126].

Raman thermal measurement technology, with its non-contact, high spatial resolution, and multi-physical quantity synchronous analysis capabilities, has become an important supplementary tool for interface thermal characterization, particularly suitable for interface thermal transport studies of low-dimensional materials (such as graphene, MoS_2_, etc.) and heterojunctions [111,123]. Unlike the TDTR method (which requires a metal transducer to be deposited), Raman measurement technology directly measures temperature through the interaction of light and materials, avoiding the introduction of external interface thermal resistance. There have been numerous reports on the use of Raman technology to measure interface thermal conductivity information in heterostructures. For example, Yalon et al. [124] combined Raman thermometry and scanning thermal microscopy to achieve high spatial resolution ITC measurements, as shown in Figure 9a–c. The Raman spectra of HfO_2_, TiO_2_, and Ge_2_Sb_2_Te_5_ (GST) thin films as a function of temperature were reported, and an interface thermal resistance of 28 ± 8 m^2^K/GW was found at the GST/SiO_2_ interface. Deng et al. [111] conducted interface heat conduction simulations of MoS_2_ supported on fused quartz using frequency domain energy transfer Raman (FET-Raman) and discussed the effects of the time domain and spatial domain on interface thermal resistance measurements. The report points out that for silicon dioxide/silicon substrates, an oxide layer thicker than 1000 nm can ensure the measurement sensitivity of interface thermal conductivity. For two-dimensional heterostructures, Zhang et al. [125] revealed the thermal transport properties of the WS_2_/Graphene two-dimensional heterostructure interface based on Raman and photoluminescence (PL) methods, as shown in Figure 9d–f. The results show that as the interlayer spacing increases, the phonon-dominated interfacial heat conduction at the WS_2_/Graphene interface further decreases until the air-dominated interfacial heat conduction increases again. Li et al. [126] developed a completely non-contact laser flash Raman testing method, with the testing device illustrated in Figure 9g, which can simultaneously measure the thermal contact resistance and thermal conductivity at the junction of a single carbon fiber (CF). Measurement results show that the laser absorption rate of CF with a diameter of 11 mm is 0.12 ± 0.03, the thermal conductivity of a single CF is approximately 200 W/mK, and the thermal contact resistance of CF/CF is (2.98 ± 0.92) × 10^5^ K/W. Yuan et al. [123] systematically studied the ITC between molybdenum disulfide (MoS_2_) and crystalline silicon (c-Si) interfaces using Raman spectroscopy measurement technology. The results show that the ITC at room temperature increases with the number of MoS_2_ layers, from 0.974 MW/m^2^K to 68.6 MW/m^2^K. Thicker samples have higher ITC values, indicating better interface contact between the film and the substrate, thereby improving the interface energy coupling accordingly. In addition, MD numerical simulations were compared with experimental measurement results for analysis.

Although Raman measurement technology can accurately measure the interface heat transfer characteristics of heterogeneous structures by utilizing its high spatial resolution, it is worth noting that Raman measurement technology is significantly material-dependent and is only applicable to materials with obvious Raman signals (such as semiconductors, carbon materials, two-dimensional materials, etc.). The Raman signals of metals and certain non-polar materials (such as SiC) are extremely weak and difficult to detect.

### 3.3. Femtosecond Laser Pump-Probe Technique

**Figure 10:**
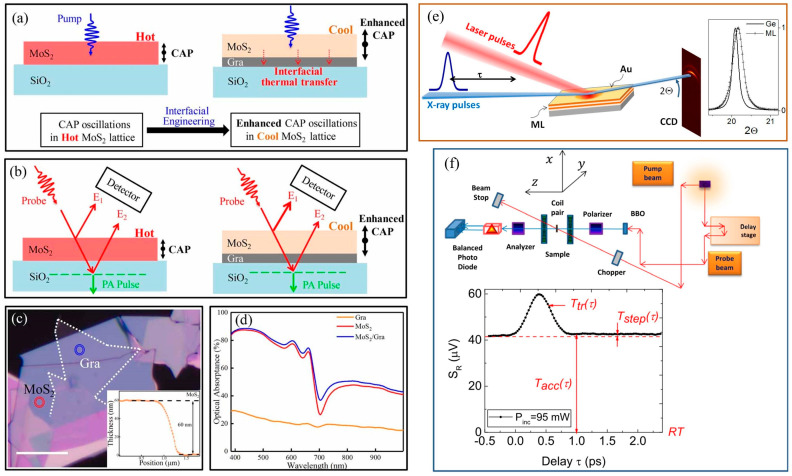
Femtosecond laser pump-probe technique applied to interface thermal conductivity measurement of heterostructures. (**a**) Enhanced coherent phonon (CAP) oscillations due to interfacial heat transfer in MoS_2_/graphene heterostructures [128]. (**b**) CAP and picosecond acoustic detection solutions [128]. (**c**) Optical microscope image of MoS_2_/graphene heterostructure [128]. (**d**) Optical absorbance of different material layers in heterostructures [128]. (**e**) A laser pump and X-ray probe device scheme for measuring cooling kinetics with a time resolution of 100 ps [112]. (**f**) Sketch of the pump-probe setup. The inset shows that the response of the sample is symmetric in the applied field [131].

Femtosecond laser pump-probe technology can directly observe non-equilibrium heat transfer at micro–nano interfaces, excite non-equilibrium hot carriers (electrons, phonons) near the interface, and track their relaxation process through the time delay of the detection signal. Direct measurement of the transient behavior of hot carriers crossing the interface can resolve ultrafast mechanisms such as phonon–electron coupling and interface scattering [112,127,128,129,130,131]. For example, Xu et al. [128] demonstrated the significant mediating role of phonon scattering in MoS_2_/graphene/glass heterostructures through femtosecond laser pump-probe measurements, as shown in Figure 10a–d. The results provide insights into manipulating coherent phonons and picosecond phonon pulses through interface engineering. Traditional thermal probes (such as thermocouples) introduce additional contact thermal resistance, whereas the femtosecond laser pump-probe method does not require physical contact, making it particularly suitable for fragile or nanostructured samples. In addition, thanks to its ultra-fast, high-resolution, and non-contact characteristics, it can be used to study the impact of micro–nano interface engineering on heat transfer at heterogeneous structure interfaces. Plech et al. [112] determined the interfacial thermal resistance of Si-based heterostructures by transient pump-probe detection of laser-induced heating dissipation, as illustrated in Figure 10e. Detailed modeling of cooling kinetics using the Laplace domain method allows the effects of thermal conductivity and interfacial thermal resistance to be distinguished, as well as basic depth information. Hopkins et al. [127] used pump-probe technology to measure the ITC of the chromium/silicon heterointerface region. The ITC data for 50 nm chromium/silicon was reported, and compared to samples that underwent reverse cutting (ITC = 113 MW/m^2^K), samples that were not prepared by reverse cutting (ITC = 178 MW/m^2^K) had an interface thermal conductivity that was approximately 60% higher. Ma et al. [129] used a backside pumping-surface detection transient thermal reflection method experiment to study the heat transfer process of femtosecond pulse laser heating of metal thin films. The theoretical prediction curves and experimental measurement results were in good agreement, verifying the correctness of the theoretical model. Based on this model, the electron–phonon coupling coefficient of gold thin films and the interfacial thermal conductivity of gold/glass (690 MW/m^2^K) and gold/silicon carbide (330 MW/m^2^K) heterostructures were measured. Hoveyda et al. [131] used pump-probe experiments and polarization microscopy to study temperature and heat flow in metallic magnetic superlattices on glass substrates. A schematic diagram of the device is shown in Figure 10f. The demagnetization mode is reproduced using Green’s function, which includes interfacial thermal conductivity.

It is worth noting that after femtosecond laser excitation, the electron–phonon system is in a highly non-equilibrium state (e.g., the electron temperature is much higher than the phonon temperature), and complex theoretical models (e.g., the two-temperature model and the Boltzmann transport equation) are required to fit the data. Errors in model assumptions (such as coupling coefficients and boundary conditions) will directly affect the results of heat conduction calculations. In addition, the spatial resolution of pump-probe technology is limited by the laser wavelength (typically greater than several hundred nanometers), making it difficult to directly resolve sub-nanometer-scale interface defects or chemical bonding details.

### 3.4. Challenges and Development Trends in ITC Measurement Technology

There are still a few key challenges in measuring thermal conductivity at the micro–nano interface. From a technical perspective, existing methods have obvious limitations in terms of flux measurement, applicable environments, and sample preparation. In addition, most high-resolution technologies require an ultra-high-vacuum environment, which is difficult to replicate in actual working conditions. In terms of theoretical analysis, it remains difficult to accurately extract interface thermal conductivity parameters from measurement signals, especially for multi-layer heterogeneous structures. Taking TDTR technology as an example, its data processing requires assumptions about the thermal properties of known materials, which often introduces significant errors in actual heterogeneous interface measurements. In addition, there is still a lack of effective means for characterizing interface thermal conductivity under dynamic conditions, such as the transient interface thermal behavior during high-frequency switching processes, which is difficult to capture accurately. Electro-thermal coupling (such as thermoelectric materials) and force-thermal coupling (such as flexible devices under pressure) can cause dynamic changes in thermal resistance. Addressing these challenges requires the development of in situ, real-time, multi-parameter, collaborative measurement technologies. It is worth noting that the measurement results obtained using different methods (TDTR, Raman measurement, 3ω method, femtosecond laser pump-probe technique, etc.) vary greatly, and there is a lack of uniform interface thermal resistance definitions and calibration standards.

Current experimental studies on the thermal resistance of heterostructure interfaces have significant limitations in terms of characterization techniques, data interpretation, and engineering relevance. Mainstream techniques (such as TDTR and FDTR) use laser spot sizes (1–10 μm) that are much larger than the interface feature size (<10 nm), resulting in measured values that are macro-equivalent thermal resistances. The contact pressure (>100 nN) of the scanning thermal microscope (SThM) probe induces interface deformation, causing an increase in the measured thermal resistance of van der Waals structures such as graphene/h-BN. In addition, steady-state measurement methods (such as the 3ω method) completely ignore frequency-dependent thermal transport characteristics, leading to a systematic underestimation of the thermal resistance at the interface of wide bandgap semiconductors (GaN). Current research has fallen into a vicious cycle where the higher the measurement accuracy, the lower the engineering relevance. The breakthrough lies in shifting from pursuing measurements of the intrinsic properties of isolated interfaces to establishing a comprehensive evaluation system covering the entire chain from preparation to measurement to service.

## 4. Conclusions and Perspectives

### 4.1. Summary of Current Research Status

This paper provides a systematic review of the current state of research on thermal resistance at the interfaces of heterostructures in micro–nano power devices. It discusses the significant impact of interface thermal resistance on device performance. A detailed analysis of the formation mechanism and influencing factors of interface thermal resistance was conducted, including material properties, interface structure, and temperature factors. Interface thermal resistance mainly originates from the scattering and energy conversion of phonons/electrons at the interface. In micro–nano power devices, the phonon density of states and dispersion relations of the materials in the heterostructure determine the characteristics of the thermal carriers. This paper summarizes the current main experimental measurement techniques and theoretical calculation methods, and compares the advantages and disadvantages of various techniques. Currently, the main techniques for measuring thermal resistance at micro- and nanoscale interfaces include time domain thermal reflectometry, frequency domain thermal reflectometry (FDTR), the 3ω method, transient pump-probe technology, and Raman thermal measurement. In terms of theoretical calculations, molecular dynamics simulations and the Boltzmann transport equation are the main methods used to study interfacial thermal resistance. Molecular dynamics can simulate heat conduction processes at the atomic scale and directly calculate temperature jumps and heat flows at interfaces, but the calculations are computationally intensive and limited by the accuracy of the potential function. Researchers have proposed various strategies for controlling interface thermal resistance. Interface engineering is the most direct method, which optimizes heat conduction by controlling interface roughness, introducing transition layers, or designing gradient structures. Emerging control methods also include interface chemical bonding control and strain engineering. Strong chemical bonds formed through surface functionalization can enhance interface coupling, while applying appropriate strain can regulate the phonon characteristics of materials. Specifically, we explored the effects of phonon/magneton quantization on ITC in ultra-low-temperature or topological interfaces and established standards for uncertainty analysis in experimental measurements (e.g., laser spot size calibration, thermal model error correction) to improve data comparability. Furthermore, we constructed a micro–nano interface heat transport database (material combinations, interface structures, ITC values) and used machine learning to predict the interface thermal conductivity of unknown systems, thereby guiding experimental design.

### 4.2. Future Challenges and Prospects

Significant progress has been made in the study of thermal resistance at the interfaces of heterostructures in micro–nano power devices, but numerous challenges remain. Future research should focus on the following areas: optimizing heat transfer at interfaces through atomic engineering, developing measurement techniques with higher spatio-temporal resolution, establishing more accurate multiscale theoretical models, exploring novel interface structures and material combinations, and investigating interface thermal transport properties under extreme conditions (e.g., high pressure, strong fields). These studies will provide a more scientific basis for thermal management design in micro–nano power devices.

#### 4.2.1. Atomic-Level Interface Engineering

**Figure 11:**
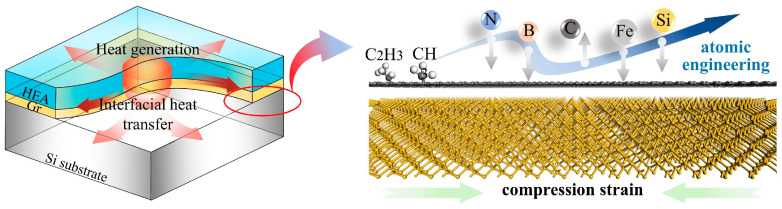
Schematic representation of atomic-level engineering optimized interface heat transfer in a heterostructure [31].

Atomic-level interface engineering has emerged as a cutting-edge approach for regulating the thermal resistance at the interfaces of heterostructures in micro- and nanoscale power devices. Passivation treatment involves introducing single-atom or molecular-layer-thick modification materials (such as sulfides, oxides, etc.) at the interface, effectively reducing the density of interface states and improving phonon coupling efficiency. Texturing technology, on the other hand, precisely controls the atomic arrangement at the interface to form specific crystal orientation relationships, such as epitaxial growth of nano-diffraction gratings or superlattice structures, which can significantly enhance phonon transmission efficiency. A schematic of the atomic-level engineering of the interface is shown in Figure 11.

#### 4.2.2. Machine Learning-Assisted Material Design and Optimization

**Figure 12:**
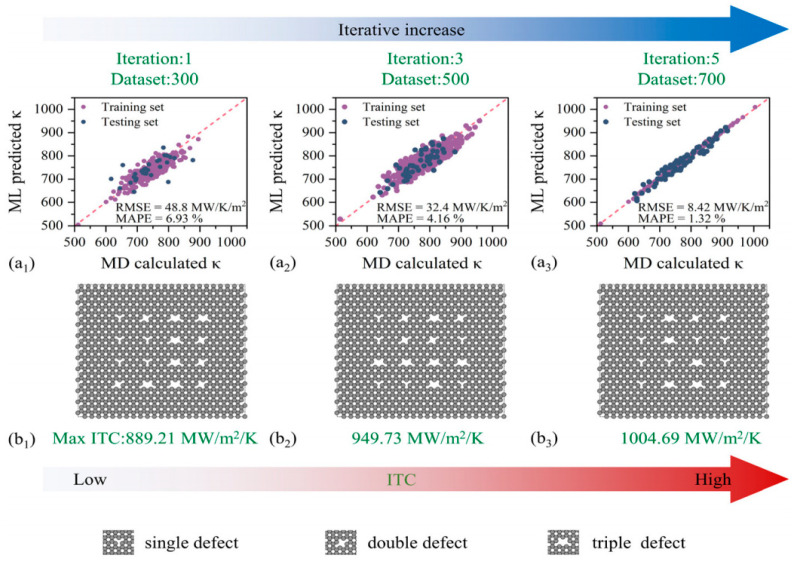
Schematic representation of atomic-level engineering optimized interface heat transfer in a heterostructure [132].

Machine learning methods show great potential in the study of interface thermal resistance. The schematic diagram is shown in Figure 12. By establishing a database linking material properties (lattice constants, phonon density of states, Debye temperature, etc.) to interface thermal resistance, deep learning models can predict the thermal transport properties of novel heterostructure combinations. Additionally, generative adversarial networks (GANs) can be used to reverse-engineer interface structures with specific thermal properties, providing innovative ideas for new material development. However, current machine learning methods face challenges such as insufficient high-quality training data and poor interpretability of physical mechanisms.

#### 4.2.3. Electrical–Thermal–Mechanical Multi-Physics Coupling Design

**Figure 13:**
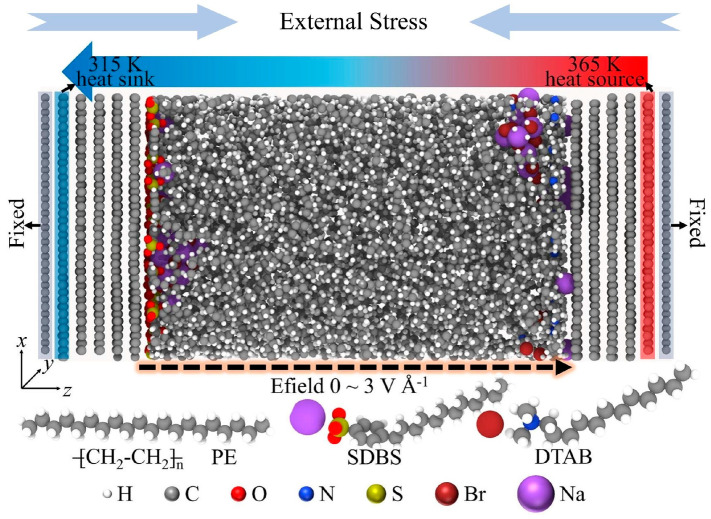
Schematic diagram of the interface heat transfer considering the electrical–thermal–mechanical multi-physics coupling case [133].

Electrical–thermal–mechanical multi-physics coupling design represents a new paradigm for addressing interface thermal resistance issues under actual device operating conditions, as illustrated in Figure 13. During the operation of power devices, the distribution of electric fields influences carrier–phonon interactions, thereby altering interface thermal transport characteristics. The multi-physics coupling model comprehensively considers the mutual influences of factors such as Joule heating, thermoelectric effects, and thermal expansion, enabling more accurate predictions of interface thermal behavior under actual operating conditions. Based on this coupling design philosophy, it is anticipated that intelligent power device structures with adaptive thermal management capabilities can be developed.

## Figures and Tables

**Table 1:** The calculation results of ITC for typical metal–semiconductor heterostructures at room temperature.

Typical Heterostructures	Computational Method	Simulated Values of ITC
Cu/Diamond	NEMD	35–48 MW/m^2^K [62,73]
Al/Diamond	MLMD	284 MW/m^2^K [70]
Al/Si	NEMD	350 MW/m^2^K [67]
Cu/Si	NEMD	600 MW/m^2^K [74]
Cu/Si	TT-MD	357 MW/m^2^K [75]
Ge/Si	NEMD	410 MW/m^2^K [44]
Al/SiC	MMM	92 MW/m^2^K [76]
Al/GaAs	AMM	420 MW/m^2^K [76]
Al/GaN	AMM	440 MW/m^2^K [76]
Cu/SWCNT	NEMD	32 MW/m^2^K [71]
Al/SWCNT	NEMD	48 MW/m^2^K [71]

Notice: TT-MD is the dual-temperature molecular dynamics simulation, and AMM is the acoustic mismatch model. MMM is the modified mismatch model.

**Table 3:** The calculation results of the ITC for novel low-dimensional heterostructures at room temperature.

Typical Heterostructures	Computational Method	Simulated Values of ITC
vdW graphene/silene	TTP	11.74 MW/m^2^K [104]
vdW MoS_2_/a-SiO_2_	TTP	5.6 MW/m^2^K [105]
vdW graphene/HEA	TTP	9.5–11.1 MW/m^2^K [64]
vdW graphene/MoS_2_	NEMD	13.8 MW/m^2^K [86]
vdW MoS_2_/MoS_2_	NEMD	44.48 MW/m^2^K [86]
vdW graphene/graphene	NEMD	212.65 MW/m^2^K [86]
In-plane graphene/h-BN	NEMD	4.3 GW/m^2^K [58]
In-plane graphene/graphane	RNEMD	22 GW/m^2^K [106]
In-plane graphene/MoS_2_	NEMD	0.225–0.25 GW/m^2^K [107]

Notice: TTP represents the transient thermal pulse method. RNEMD represents reverse non-equilibrium molecular dynamics.

## Data Availability

The raw/processed data required to reproduce these findings cannot be shared at this time as the data also forms part of an ongoing study.

## References

[1] Liu Q., Yu W., Luo H., Ren X., Shen S. (2020). Tuning thermal resistance of SiC crystal/amorphous layered nanostructures via changing layer thickness. Comput. Mater. Sci..

[2] Zhao H., Yang X., Wang C., Lu R., Zhang T., Chen H., Zheng X. (2023). Progress in thermal rectification due to heat conduction in micro/nano solids. Mater. Today Phys..

[3] Awais M., Chen X., Hong Z., Wang Q., Shi Y., Meng F.-B., Dai C., Paramane A. (2022). Synergistic effects of Micro-hBN and core-shell Nano-TiO_2_@SiO_2_ on thermal and electrical properties of epoxy at high frequencies and temperatures. Compos. Sci. Technol..

[4] Weituschat L.M., Dickmann W., Guimbao J., Ramos D., Kroker S., Postigo P.A. (2020). Photonic and Thermal Modelling of Microrings in Silicon, Diamond and GaN for Temperature Sensing. Nanomaterials.

[5] Ramos C., Maelo Ferrer A., Santana G., Calvo Mola C., Chaviano M., Fonseca D., González Y., Ruediger A., de Melo O., Sánchez M. (2025). Surface photovoltage spectroscopy for texture and passivation processes monitoring in black silicon solar cells. Sol. Energy Mater. Sol. Cells.

[6] Lee P.-Y., Chen T.-C., Huang J.-Y., Hsieh H.-L., Jang J.S.-C. (2014). Enhancement of the thermoelectric performance in nano-/micro-structured p-type Bi0.4Sb1.6Te3 fabricated by mechanical alloying and vacuum hot pressing. J. Alloys Compd..

[7] Pathumudy R.D., Prabhu K.N. (2021). Thermal interface materials for cooling microelectronic systems: Present status and future challenges. J. Mater. Sci. Mater. Electron..

[8] Kapitza P.L. (1941). Heat Transfer and Superfluidity of Helium II. Phys. Rev..

[9] Singh P., Seong M., Sinha S. (2013). Detailed consideration of the electron-phonon thermal conductance at metal-dielectric interfaces. Appl. Phys. Lett..

[10] Tian S., Xu Z., Wu S., Luo T., Xiong G. (2022). Anisotropically tuning interfacial thermal conductance between graphite and poly(ethylene oxide) by lithium-ion intercalation: A molecular dynamics study. Int. J. Heat Mass Transf..

[11] Chen X.-K., Zeng Y.-J., Chen K.-Q. (2020). Thermal Transport in Two-Dimensional Heterostructures. Front. Mater..

[12] Wang J.P., Shen Y.J., Yang P. (2023). Multilayered Graphene/ZnO heterostructure interfaces to improve thermal transfer. Compos. Commun..

[13] Goni M., Patelka M., Ikeda S., Hartman T., Sato T., Schmidt A.J. (2018). A technique to measure the thermal resistance at the interface between a micron size particle and its matrix in composite materials. J. Appl. Phys..

[14] De Mey G., Pilarski J., Wójcik M., Lasota M., Banaszczyk J., Vermeersch B., Napieralski A. (2009). Influence of interface materials on the thermal impedance of electronic packages. Int. Commun. Heat Mass Transf..

[15] Gardea F., Naraghi M., Lagoudas D. (2013). Effect of Thermal Interface on Heat Flow in Carbon Nanofiber Composites. ACS Appl. Mater. Interfaces.

[16] Hahn K.R., Puligheddu M., Colombo L. (2015). Thermal boundary resistance at Si/Ge interfaces determined by approach-to-equilibrium molecular dynamics simulations. Phys. Rev. B.

[17] Stevens R.J., Zhigilei L.V., Norris P.M. (2007). Effects of temperature and disorder on thermal boundary conductance at solid–solid interfaces: Nonequilibrium molecular dynamics simulations. Int. J. Heat Mass Transf..

[18] Huang M.-J., Tsai P.-K. (2022). The size effect on the interfacial thermal resistances of sandwich structures. Int. J. Heat Mass Transf..

[19] Liu M., Zhang H., Wu Y., Wang D., Pan L. (2024). Effect of functionalization on thermal conductivity of hexagonal boron nitride/epoxy composites. Int. J. Heat Mass Transf..

[20] Yang H.Y., Tang Y.Q., Yang P. (2023). Building efficient interfacial property with graphene heterogeneous interface. Int. J. Mech. Sci..

[21] Serebryakova M.A., Zaikovskii A.V., Sakhapov S.Z., Smovzh D.V., Sukhinin G.I., Novopashin S.A. (2017). Thermal conductivity of nanofluids based on hollow γ-Al_2_O_3_ nanoparticles, and the influence of interfacial thermal resistance. Int. J. Heat Mass Transf..

[22] Glazov A.L., Kalinovskii V.S., Muratikov K.L. (2018). Heat transfer through soldered and bonded joints of multilayer semiconductor devices studied by laser photothermal beam-deflection method. Int. J. Heat Mass Transf..

[23] Jesudason C.G. (2017). Fourier heat conduction characteristics of a lattice chain arising from considerations in intermolecular potentials and the Second law. Int. J. Therm. Sci..

[24] Péraud J.-P.M., Hadjiconstantinou N.G. (2016). Extending the range of validity of Fourier’s law into the kinetic transport regime via asymptotic solution of the phonon Boltzmann transport equation. Phys. Rev. B.

[25] Candela D., Walsworth R.L. (2007). Understanding the breakdown of Fourier’s law in granular fluids. Am. J. Phys..

[26] Cheng Z., Koh Y.R., Ahmad H., Hu R., Shi J., Liao M.E., Wang Y., Bai T., Li R., Lee E. (2020). Thermal conductance across harmonic-matched epitaxial Al-sapphire heterointerfaces. Commun. Phys..

[27] O’Brien P.J., Shenogin S., Liu J., Chow P.K., Laurencin D., Mutin P.H., Yamaguchi M., Keblinski P., Ramanath G. (2012). Bonding-induced thermal conductance enhancement at inorganic heterointerfaces using nanomolecular monolayers. Nat. Mater..

[28] Sadasivam S., Waghmare U.V., Fisher T.S. (2015). Electron-phonon coupling and thermal conductance at a metal-semiconductor interface: First-principles analysis. J. Appl. Phys..

[29] Dai W., Wang Y., Li M., Chen L., Yan Q., Yu J., Jiang N., Lin C.T. (2024). 2D Materials-Based Thermal Interface Materials: Structure, Properties, and Applications. Adv. Mater..

[30] Kudryashov S.I., Gakovic B., Danilov P.A., Petrovic S.M., Milovanovic D., Rudenko A.A., Ionin A.A. (2018). Single-shot selective femtosecond laser ablation of multi-layered Ti/Al and Ni/Ti films: “Cascaded” heat conduction and interfacial thermal effects. Appl. Phys. Lett..

[31] Shen Y., Yang H., Cao K., Yang P. (2025). Interlayer surface modification modulating thermal transport at Si/Gr/HEA heterostructure interfaces. Int. J. Therm. Sci..

[32] Liu Z., Feng Y., Li H., Cao N., Qiu L. (2024). Quantitative analysis of interface heat transport at the Si_3_N_4_/SiO_2_ van-der Waals point contact. Int. J. Heat Mass Transf..

[33] Li Y., Mehra N., Ji T., Zhu J. (2018). Realizing the nanoscale quantitative thermal mapping of scanning thermal microscopy by resilient tip–surface contact resistance models. Nanoscale Horiz..

[34] Abs da Cruz C., Chantrenne P., Gomes de Aguiar Veiga R., Perez M., Kleber X. (2013). Modified embedded-atom method interatomic potential and interfacial thermal conductance of Si-Cu systems: A molecular dynamics study. J. Appl. Phys..

[35] Li D., Shen Y., Yang P. (2021). N-Doped and P-Doped Graphene on MgO (111): A First-Principles Study. Adv. Eng. Mater..

[36] Kononenko O., Brzhezinskaya M., Zotov A., Korepanov V., Levashov V., Matveev V., Roshchupkin D. (2022). Influence of numerous Moiré superlattices on transport properties of twisted multilayer graphene. Carbon.

[37] Brzhezinskaya M., Kononenko O., Matveev V., Zotov A., Khodos I.I., Levashov V., Volkov V., Bozhko S.I., Chekmazov S.V., Roshchupkin D. (2021). Engineering of Numerous Moiré Superlattices in Twisted Multilayer Graphene for Twistronics and Straintronics Applications. ACS Nano.

[38] Chen J., Xu X., Zhou J., Li B. (2022). Interfacial thermal resistance: Past, present and future. Rev. Mod. Phys..

[39] Zhang P., Yuan P., Jiang X., Zhai S., Zeng J., Xian Y., Qin H., Yang D. (2017). A Theoretical Review on Interfacial Thermal Transport at the Nanoscale. Small.

[40] Giri A., Hopkins P.E. (2019). A Review of Experimental and Computational Advances in Thermal Boundary Conductance and Nanoscale Thermal Transport across Solid Interfaces. Adv. Funct. Mater..

[41] Yang B., Tang Y., Xin Z., Zheng H., Qi D., Zhang N., Tang Y., Wu X. (2024). Modulation of the interfacial thermal resistances of the w-AlN/Graphene/3C-SiC interface by nanoscale nonplanar feature structures. Appl. Surf. Sci..

[42] Shen Y., Li D., Cheng Z., Tang Y., Yang P. (2024). Coupling field optimization to improve the thermal transport of Gr/h-BN heterostructure. Diam. Relat. Mater..

[43] Bazrafshan S., Rajabpour A. (2017). Thermal transport engineering in amorphous graphene: Non-equilibrium molecular dynamics study. Int. J. Heat Mass Transf..

[44] Feng T., Zhong Y., Shi J., Ruan X. (2019). Unexpected high inelastic phonon transport across solid-solid interface: Modal nonequilibrium molecular dynamics simulations and Landauer analysis. Phys. Rev. B.

[45] Shan S., Zhang Z., Volz S., Chen J. (2024). Phonon mode at interface and its impact on interfacial thermal transport. J. Phys. Condens. Matter.

[46] Stoneley R. (1997). Elastic waves at the surface of separation of two solids. Proc. R. Soc. London Ser. A Contain. Pap. A Math. Phys. Character.

[47] Ren S.-F., Chu H., Chang Y.-C. (1988). Anisotropy of optical phonons and interface modes in GaAs-AlAs superlattices. Phys. Rev. B.

[48] Martinez M., Cardani L., Casali N., Cruciani A., Pettinari G., Vignati M. (2019). Measurements and Simulations of Athermal Phonon Transmission from Silicon Absorbers to Aluminum Sensors. Phys. Rev. Appl..

[49] Yang N., Luo T., Esfarjani K., Henry A., Tian Z., Shiomi J., Chalopin Y., Li B., Chen G. (2015). Thermal Interface Conductance Between Aluminum and Silicon by Molecular Dynamics Simulations. J. Comput. Theor. Nanosci..

[50] Ma D., Zhang G., Zhang L. (2020). Interface thermal conductance between β-Ga2O3 and different substrates. J. Phys. D Appl. Phys..

[51] Merabia S., Termentzidis K. (2012). Thermal conductance at the interface between crystals using equilibrium and nonequilibrium molecular dynamics. Phys. Rev. B.

[52] Polat S. (2022). Theoretical modeling and optimization of interface design to improve thermal conductivity in Mg-Dia composites. Ceram. Int..

[53] Fourier J.B.J. (1822). Th’eorie Analytique de la Chaleur.

[54] Poisson S.-D. (1835). Th’eorie Math’ematique de la Chaleur.

[55] Wang Z., Sun F., Liu Z., Zheng L., Wang D., Feng Y. (2023). Regulated Thermal Boundary Conductance between Copper and Diamond through Nanoscale Interfacial Rough Structures. ACS Appl. Mater. Interfaces.

[56] Dickey J.M., Paskin A. (1969). Computer Simulation of the Lattice Dynamics of Solids. Phys. Rev..

[57] Xu X.Y., Shen Y.J., Yang P. (2023). Building efficient thermal transport at graphene/polypropylene interfaces by non-covalent functionalized graphene. Phys. Lett. A.

[58] Liang T., Zhou M., Zhang P., Yuan P., Yang D.G. (2020). Multilayer in-plane graphene/hexagonal boron nitride heterostructures: Insights into the interfacial thermal transport properties. Int. J. Heat Mass Transf..

[59] Wang Q., Zhang J., Xiong Y., Li S., Chernysh V., Liu X. (2023). Atomic-Scale Surface Engineering for Giant Thermal Transport Enhancement Across 2D/3D van der Waals Interfaces. ACS Appl. Mater. Interfaces.

[60] Wang B.C., Cao Q., Shao W., Cui Z. (2022). Effect of vacancy defects on the heat transfer coefficient of partially stacked graphene sheets. J. Mater. Sci..

[61] Das P., Paul P., Hassan M., Monjur Morshed A.K.M., Paul T.C. (2025). Interfacial thermal resistance in stanene/hexagonal boron nitride van der Waals heterostructures: A molecular dynamics study. Comput. Mater. Sci..

[62] Liao J., Zhang M., Yang D., He Z., Liu Y., Li L. (2025). The interfacial roughness dependence of Cu/diamond thermal boundary conductance: A molecular dynamics study. Diam. Relat. Mater..

[63] Zhu Y., Yin E., Luo W., Li Q. (2025). Multiscale thermal analysis of diamond/Cu composites for thermal management applications by combining lattice Boltzmann and finite element methods. Int. J. Therm. Sci..

[64] Shen Y., Guo J., Tang Y., Yang P. (2025). Gr/HEA-FexNiCrCoCu interface getting excellent thermal transport. Intermetallics.

[65] Li Y., Zhou H., Wu C., Yin Z., Liu C., Liu J., Shi Z. (2023). Interfacial Characterization and Thermal Conductivity of Diamond/Cu Composites Prepared by Liquid-Solid Separation Technique. Nanomaterials.

[66] Sinha V., Spowart J.E. (2012). Influence of interfacial carbide layer characteristics on thermal properties of copper–diamond composites. J. Mater. Sci..

[67] Xu Y., Fan H., Li Z., Zhou Y. (2023). Signatures of anharmonic phonon transport in ultrahigh thermal conductance across atomically sharp metal/semiconductor interface. Int. J. Heat Mass Transf..

[68] Hohensee G.T., Wilson R.B., Cahill D.G. (2015). Thermal conductance of metal–diamond interfaces at high pressure. Nat. Commun..

[69] Cheng C., Ma S.Y., Wang S.Q. (2023). The role of phonon anharmonicity on the structural stability and phonon heat transport of CrFeCoNiCux high-entropy alloys at finite temperatures. J. Alloys Compd..

[70] Adnan K.Z., Neupane M.R., Feng T. (2024). Thermal boundary conductance of metal–diamond interfaces predicted by machine learning interatomic potentials. Int. J. Heat Mass Transf..

[71] Shenogin S., Gengler J., Roy A., Voevodin A.A., Muratore C. (2013). Molecular dynamics studies of thermal boundary resistance at carbon–metal interfaces. Scr. Mater..

[72] Cai X., Li H., Zhang J., Ma T., Wang Q. (2025). Mechanism of interfacial thermal resistance variation in diamond/Cu/CNT tri-layer during thermal cycles. Int. J. Therm. Sci..

[73] Wu K., Zhang L., Li F., Sang L., Liao M., Tang K., Ye J., Gu S. (2024). Enhancement of interfacial thermal conductance by introducing carbon vacancy at the Cu/diamond interface. Carbon.

[74] Xu Y., Cao B.-Y., Zhou Y. (2024). Near-interface effects on interfacial phonon transport: Competition between phonon-phonon interference and phonon-phonon scattering. Int. J. Heat Mass Transf..

[75] Lu Z., Wang Y., Ruan X. (2016). Metal/dielectric thermal interfacial transport considering cross-interface electron-phonon coupling: Theory, two-temperature molecular dynamics, and thermal circuit. Phys. Rev. B.

[76] Zong Z.-C., Pan D.-K., Deng S.-C., Wan X., Yang L.-N., Ma D.-K., Yang N. (2023). Mixed mismatch model predicted interfacial thermal conductance of metal/semiconductor interface. Acta Phys. Sin..

[77] Di Ventra M., Berthod C., Binggeli N. (2005). Heterovalent interlayers and interface states: Anab initiostudy ofGaAs∕Si∕GaAs(110) and (100) heterostructures. Phys. Rev. B.

[78] Li Y., Chen W.-H. (2006). Numerical simulation of electrical characteristics in nanoscale Si/GaAs MOSFETs. J. Comput. Electron..

[79] Mi Z., Bianucci P. (2012). When self-organized In(Ga)As/GaAs quantum dot heterostructures roll up: Emerging devices and applications. Curr. Opin. Solid State Mater. Sci..

[80] Qiu Y., Qiu X., Guo X., Wang D., Sun L. (2017). Thermal Analysis of Si/GaAs Bonding Wafers and Mitigation Strategies of the Bonding Stresses. Adv. Mater. Sci. Eng..

[81] Kim K., Jang J., Kim H. (2021). Negative differential resistance in Si/GaAs tunnel junction formed by single crystalline nanomembrane transfer method. Results Phys..

[82] Vega-Flick A., Jung D., Yue S., Bowers J.E., Liao B. (2019). Reduced thermal conductivity of epitaxial GaAs on Si due to symmetry-breaking biaxial strain. Phys. Rev. Mater..

[83] Xue J., Li F., Fan A., Ma W., Zhang X. (2025). Optimizing interfacial thermal resistance in GaN/AlN heterostructures: The impact of AlN layer thickness. Int. J. Heat Mass Transf..

[84] Wu S., Kang D., Yu X., Dai J. (2024). Thermal transport across armchair–zigzag graphene homointerface. Appl. Phys. Lett..

[85] Wu D., Ding H., Fan Z.Q., Jia P.Z., Xie H.Q., Chen X.K. (2022). High interfacial thermal conductance across heterogeneous GaN/graphene interface. Appl. Surf. Sci..

[86] Ding Z.W., Pei Q.-X., Jiang J.-W., Huang W.X., Zhang Y.-W. (2016). Interfacial thermal conductance in graphene/MoS2 heterostructures. Carbon.

[87] Gu L., Li Y., Shen Y., Yang R.-Y., Ma H.-P., Sun F.Y., Zuo Y., Tang Z., Yuan Q., Jiang N. (2024). A strategy for enhancing interfacial thermal transport in Ga2O3-diamond composite structure by introducing an AlN interlayer. Nano Energy.

[88] Zhao X., Qu Y., Deng N., Yuan J., Du L., Hu W., Wang H. (2024). Structural and phonon transport analysis of surface-activated bonded SiC-SiC homogenous interfaces. Appl. Surf. Sci..

[89] Liu Y.L., Qiu L., Liu J.L., Feng Y.H. (2023). Enhancing thermal transport across diamond/graphene heterostructure interface. Int. J. Heat Mass Transf..

[90] Yu X., Li Y., He R., Wen Y., Chen R., Xu B., Gao Y. (2024). Mechanical Regulation to Interfacial Thermal Transport in GaN/Diamond Heterostructures for Thermal Switch. Nanoscale Horiz..

[91] Rajabpour A., Mortazavi B., Mirchi P., El Hajj J., Guo Y., Zhuang X., Merabia S. (2025). Accurate estimation of interfacial thermal conductance between silicon and diamond enabled by a machine learning interatomic potential. Int. J. Therm. Sci..

[92] Liu Y., Qiu L., Wang Z., Li H., Feng Y. (2024). Enhancing interfacial thermal transport efficiently in diamond/graphene heterostructure by involving vacancy defects. Compos. Part A Appl. Sci. Manuf..

[93] Khosravian N., Samani M.K., Loh G.C., Chen G.C.K., Baillargeat D., Tay B.K. (2013). Molecular dynamic simulation of diamond/silicon interfacial thermal conductance. J. Appl. Phys..

[94] Song C., Yang B., Wei B., Tang Y., Wu X. (2025). Mechanism analysis of strengthening interfacial thermal conductance of w-AlN/Graphene/3C-SiC typical heterostructure by graphene interlayer bonding. Ceram. Int..

[95] Wang B., Shao W., Cao Q., Cui Z. (2023). Thermal Conductivity Enhancement of Graphene/Epoxy Nanocomposites by Reducing Interfacial Thermal Resistance. J. Phys. Chem. C.

[96] Li D.B., Yang H.Y., Li L., Yang P. (2023). Numerical investigation of thermal conductivity of Cu/graphene/Cu interface. Appl. Phys. Lett..

[97] Liu F., Zhu Y., Wu R., Zou R., Zhou S., Ning H., Hu N., Yan C. (2023). Insights into the interfacial thermal transport properties of in-plane graphene/h-BN heterostructure with grain boundary. Int. J. Heat Mass Transf..

[98] Shen M., Hao Z., Song J., An M., Ying T., Xue X., Gao Y., Yang Z. (2024). Architectural and component design of CNTs/Al hierarchical composite for enhanced mechanical/thermal properties. J. Mater. Res. Technol..

[99] Meftakhutdinov R.M., Sibatov R.T. (2022). Janus Type Monolayers of S-MoSiN2 Family and Van Der Waals Heterostructures with Graphene: DFT-Based Study. Nanomaterials.

[100] Šilhavík M., Kumar P., Levinský P., Zafar Z.A., Hejtmánek J., Červenka J. (2024). Anderson Localization of Phonons in Thermally Superinsulating Graphene Aerogels with Metal-Like Electrical Conductivity. Small Methods.

[101] Yang Y.Z., Ma J., Yang J., Zhang Y.Y. (2022). Molecular Dynamics Simulation on In-Plane Thermal Conductivity of Graphene/Hexagonal Boron Nitride van der Waals Heterostructures. ACS Appl. Mater. Interfaces.

[102] Hong Y., Li L., Zeng X.C., Zhang J. (2015). Tuning thermal contact conductance at graphene-copper interface via surface nanoengineering. Nanoscale.

[103] Wu B.Y., Zhou M., Xu D.J., Liu J.J., Tang R.J., Zhang P. (2022). Interfacial thermal conductance of BP/MoS(2 )van der Waals heterostructures: An insight from the phonon transport. Surf. Interfaces.

[104] Liu B., Baimova J.A., Reddy C.D., Law A.W.-K., Dmitriev S.V., Wu H., Zhou K. (2014). Interfacial Thermal Conductance of a Silicene/Graphene Bilayer Heterostructure and the Effect of Hydrogenation. ACS Appl. Mater. Interfaces.

[105] Zhang L., Zhong Y., Qian X., Song Q., Zhou J., Li L., Guo L., Chen G., Wang E.N. (2021). Toward Optimal Heat Transfer of 2D-3D Heterostructures via van der Waals Binding Effects. ACS Appl. Mater. Interfaces.

[106] Li Y.F., Wei A.R., Datta D. (2017). Thermal characteristics of graphene nanoribbons endorsed by surface functionalization. Carbon.

[107] Liu X., Gao J., Zhang G., Zhang Y.-W. (2017). MoS2-graphene in-plane contact for high interfacial thermal conduction. Nano Res..

[108] Chen X.K., Xie Z.X., Zhou W.X., Tang L.M., Chen K.Q. (2016). Thermal rectification and negative differential thermal resistance behaviors in graphene/hexagonal boron nitride heterojunction. Carbon.

[109] Ni Y., Zhang H., Hu S., Wang H., Volz S., Xiong S. (2019). Interface diffusion-induced phonon localization in two-dimensional lateral heterostructures. Int. J. Heat Mass Transf..

[110] Hunter N., Azam N., Zobeiri H., Van Velson N., Mahjouri-Samani M., Wang X. (2022). Interface Thermal Resistance between Monolayer WSe_2_ and SiO_2_: Raman Probing with Consideration of Optical–Acoustic Phonon Nonequilibrium. Adv. Mater. Interfaces.

[111] Deng Z., Cai J., Wang G., Liu J. (2024). Interfacial thermal resistance measurement sensitivity in time and spatial domains of FET-Raman for supported 2D materials. Int. J. Heat Mass Transf..

[112] Plech A., Krause B., Baumbach T., Zakharova M., Eon S., Girmen C., Buth G., Bracht H. (2019). Structural and Thermal Characterisation of Nanofilms by Time-Resolved X-ray Scattering. Nanomaterials.

[113] Cui S., Sun F., Wang D., Zhang X., Zhang H., Feng Y. (2024). Enhancing interfacial heat conduction in diamond-reinforced copper composites with boron carbide interlayers for thermal management. Compos. Part B Eng..

[114] Liu F., Mao R., Liu Z., Du J., Gao P. (2025). Probing phonon transport dynamics across an interface by electron microscopy. Nature.

[115] Chen N., Yang K., Wang Z., Zhong B., Wang J., Song J., Li Q., Ni J., Sun F., Liu Y. (2023). Quantifying Interfacial Bonding Using Thermal Boundary Conductance at Cubic Boron Nitride/Copper Interfaces with a Large Mismatch of Phonon Density of States. ACS Appl. Mater. Interfaces.

[116] Zheng W., McClellan C.J., Pop E., Koh Y.K. (2022). Nonequilibrium Phonon Thermal Resistance at MoS_2_/Oxide and Graphene/Oxide Interfaces. ACS Appl. Mater. Interfaces.

[117] Yang S., Song H., Peng Y., Zhao L., Tong Y., Kang F., Xu M., Sun B., Wang X. (2021). Reduced thermal boundary conductance in GaN-based electronic devices introduced by metal bonding layer. Nano Res..

[118] Li R., Hussain K., Liao M.E., Huynh K., Hoque M.S.B., Wyant S., Koh Y.R., Xu Z., Wang Y., Luccioni D.P. (2024). Enhanced Thermal Boundary Conductance across GaN/SiC Interfaces with AlN Transition Layers. ACS Appl. Mater. Interfaces.

[119] Khan S., Angeles F., Wright J., Vishwakarma S., Ortiz V.H., Guzman E., Kargar F., Balandin A.A., Smith D.J., Jena D. (2022). Properties for Thermally Conductive Interfaces with Wide Band Gap Materials. ACS Appl. Mater. Interfaces.

[120] Larkin L.S., Smoyer J.L., Norris P.M. (2017). Laser repetition rate in time-domain thermoreflectance techniques. Int. J. Heat Mass Transf..

[121] Gengler J.J., Roy S., Jones J.G., Gord J.R. (2012). Two-color time-domain thermoreflectance of various metal transducers with an optical parametric oscillator. Meas. Sci. Technol..

[122] Li M., Pan K., Ge Y., Huynh K., Goorsky M.S., Fisher T.S., Hu Y. (2024). Wafer-scale bonded GaN–AlN with high interface thermal conductance. Appl. Phys. Lett..

[123] Yuan P.Y., Li C., Xu S., Liu J., Wang X.W. (2017). Interfacial thermal conductance between few to tens of layered-MoS_2_ and c-Si: Effect of MoS_2_ thickness. Acta Mater..

[124] Yalon E., Deshmukh S., Muñoz Rojo M., Lian F., Neumann C.M., Xiong F., Pop E. (2017). Spatially Resolved Thermometry of Resistive Memory Devices. Sci. Rep..

[125] Zhang R., Gan L., Zhang D., Sun H., Li Y., Ning C.Z. (2024). Extreme Thermal Insulation and Tradeoff of Thermal Transport Mechanisms between Graphene and WS2 Monolayers. Adv. Mater..

[126] Li Q.-Y., Katakami K., Ikuta T., Kohno M., Zhang X., Takahashi K. (2019). Measurement of thermal contact resistance between individual carbon fibers using a laser-flash Raman mapping method. Carbon.

[127] Hopkins P.E., Norris P.M. (2006). Thermal boundary conductance response to a change in Cr∕Si interfacial properties. Appl. Phys. Lett..

[128] Xu W., Hou F., Zhang H., Xia C., Li Z., Li Y., Xu C., Cui Q. (2024). Mediating coherent acoustic phonon oscillation of a 2D semiconductor/3D dielectric heterostructure by interfacial engineering. J. Phys. D Appl. Phys..

[129] Ma W.G., Wang H.D., Zhang X., Wang W. (2011). Theoretical and experimental study of femtosecond pulse laser heating on thin metal film. Acta Phys. Sin..

[130] Mohamad H.J., Shelford L.R., Aziz M., Al-Jarah U.A.S., Al-Saigh R., Valkass R.A.J., Marmion S., Hickey B.J., Hicken R.J. (2017). Thermally induced magnetization dynamics of optically excited YIG/Cu/Ni81Fe19 trilayers. Phys. Rev. B.

[131] Hoveyda F., Adnani M., Smadici S. (2017). Heat diffusion in magnetic superlattices on glass substrates. J. Appl. Phys..

[132] Yang H., Li L., Yang P. (2025). Deep learning-based molecular dynamics simulation reconfiguration of efficient heat energy transport of Gra/h-BN heterointerface. Appl. Phys. Lett..

[133] Sun H., Surblys D., Ohara T. (2025). Enhancement of thermal transport via electrostatic surface modification by ionic organic additives under electric fields: A molecular dynamics study. Appl. Therm. Eng..

